# Combining nanobody labeling with STED microscopy reveals input-specific and layer-specific organization of neocortical synapses

**DOI:** 10.1371/journal.pbio.3002649

**Published:** 2025-04-04

**Authors:** Yeasmin Akter, Grace Jones, Grant J. Daskivich, Victoria Shifflett, Karina J. Vargas, Martin Hruska

**Affiliations:** 1 Department of Neuroscience, Rockefeller Neuroscience Institute, West Virginia University, Morgantown, West Virginia, United States of America; 2 Department of Cell Biology, School of Medicine, University of Pittsburgh, Pittsburgh, Pennsylvania, United States of America; Harvard Medical School, UNITED STATES OF AMERICA

## Abstract

The discovery of synaptic nanostructures revealed key insights into the molecular logic of synaptic function and plasticity. Yet, our understanding of how diverse synapses in the brain organize their nano-architecture remains elusive, largely due to the limitations of super-resolution imaging in complex brain tissue. Here, we characterized single-domain camelid nanobodies for the 3D quantitative multiplex imaging of synaptic nano-organization using tau-STED nanoscopy in cryosections from the mouse primary somatosensory cortex. We focused on thalamocortical (TC) and corticocortical (CC) synapses along the apical-basal axis of layer five pyramidal neurons as models of functionally diverse glutamatergic synapses in the brain. Spines receiving TC input were larger than those receiving CC input in all layers examined. However, the nano-architecture of TC synapses varied with dendritic location. TC afferents on apical dendrites frequently contacted spines with multiple aligned PSD-95/Bassoon nanomodules of constant size. In contrast, TC spines on basal dendrites predominantly contained a single aligned nanomodule, with PSD-95 nanocluster sizes scaling proportionally with spine volume. The nano-organization of CC synapses did not change across cortical layers and resembled modular architecture defined in vitro. These findings highlight the nanoscale diversity of synaptic architecture in the brain, that is, shaped by both the source of afferent input and the subcellular localization of individual synaptic contacts.

## Abstract 2

How do diverse synapses in the brain organize their nanoarchitecture to meet their different functional requirements? This study demonstrates the use of nanobodies combined with STED imaging to study the nanoarchitecture of thalamocortical and corticocortical synapses, revealing distinct principles of synaptic nano-organization in the brain.

## Introduction

Synapses in the central nervous system are small, highly specialized cell-cell junctions designed to rapidly transfer and process information. A characteristic feature of central synapses is their remarkable structural and functional variability [1,2]. Each pyramidal neuron receives thousands of synapses with unique signaling properties that form the basis for cortical computation and information storage in the brain [3–10]. At each synapse, diverse proteins with distinct lifetimes organize at the nanoscale in an ordered manner to build active zones and post-synaptic densities (PSDs) that endow synapses with exquisite regulation of synaptic function [11–19]. Despite their importance for brain function, plasticity, and aging, how functionally diverse synapses are organized at nanoscale and how the source of afferent input might shape synaptic nano-organization is not understood.

In dendritic spines, modular nano-architecture is thought to provide the flexibility needed for dynamic processes that underlie synaptic plasticity [20–23]. Key post-synaptic components—PSD-95, AMPARs, NMDARs precisely aligned to pre-synaptic Bassoon, Vesicular Glutamate Transporter 1 (VGluT1), Synaptotagmin-1 (SYT1)—conform to this scalability in vitro [24,25]. Whether these principles underlie the organization of diverse synapses in the brain will require 3D reconstruction of molecularly identified connections in their native environment.

Pyramidal neurons in the somatosensory cortex receive functionally diverse corticocortical (CC) and thalamocortical (TC) glutamatergic innervation. Although TC synapses are the minority of cortical synapses, they are highly efficient [26–31]. While some studies suggested that TC synapses onto spiny neurons in layer 4 (L4) are multi-quantal and several-fold stronger than unitary CC synaptic connections on the same neurons, others demonstrated that TC and CC synapses in L4 are indistinguishable electrophysiologically [32–34]. Instead, clustering of TC synapses in specific dendritic domains was suggested to synchronize feedforward activity [35–37]. More recently, work using expansion microscopy in the mouse visual cortex showed that spines of L2/3 pyramidal neurons receiving TC input are smaller and weaker than neighboring CC synapses [38]. These data suggest that mechanisms regulating TC synaptic function are complex and vary depending on the cell type and cortical location. Understanding the principles of nano-organization of TC and CC synapses across layers will shed light on processing of cortical information in the brain.

Immunolabeling enables simultaneous imaging of multiple endogenous proteins, underscoring its potential to reveal the molecular complexity of synapses. However, labeling in PFA-fixed brain tissue can be challenging, especially for PSD molecules like PSD-95, NMDARs, and AMPARs, raising questions about its effectiveness in revealing synaptic nano-organization [12,39–42]. As affinity probes, single-domain nanobodies offer a promising solution to these barriers, enabling multiplexed, high-resolution imaging essential for detailed analysis of synaptic diversity [43–45].

Here, we characterized an approach for robust identification of synaptic nano-architecture by immunolabeling in brain cryosections. By combining nanobody labeling and 3D tau-STED imaging of L5 pyramidal neurons, we show that spines receiving TC input, but not those receiving CC input, exhibit variability in their modular nano-organization across cortical layers. These findings emphasize the importance of afferent input and subcellular localization in shaping synaptic nano-architecture in the brain.

## Results

### Efficient labeling of synapses by nanobodies in PFA-fixed cryosections

Accurate 3D nanoscale reconstruction of individual synapses necessitates the precise localization of pre- and post-synaptic proteins to the same synapse. However, immunolabeling PFA-fixed brain cryosections from Thy-1-YFP-H mice with antibodies targeting PSD-95 (PSDs), VGluT1 (synaptic vesicles), and Bassoon (active zones) resulted in highly variable densities of pre- and post-synaptic puncta in situ (S1A-S1D Fig). Furthermore, the colocalization of PSD-95 with either VGluT1 or Bassoon identified only a small fraction of putative excitatory synapses (<40%), significantly underestimating their number, assuming that most spines contain a functional synapse (S1E-S1G and S2 Figs) [3]. These data highlight limitations of immunolabeling in PFA-fixed samples that could hamper precise localization of trans*-*synaptic nanodomains in super-resolution approaches.

**Fig 1:**
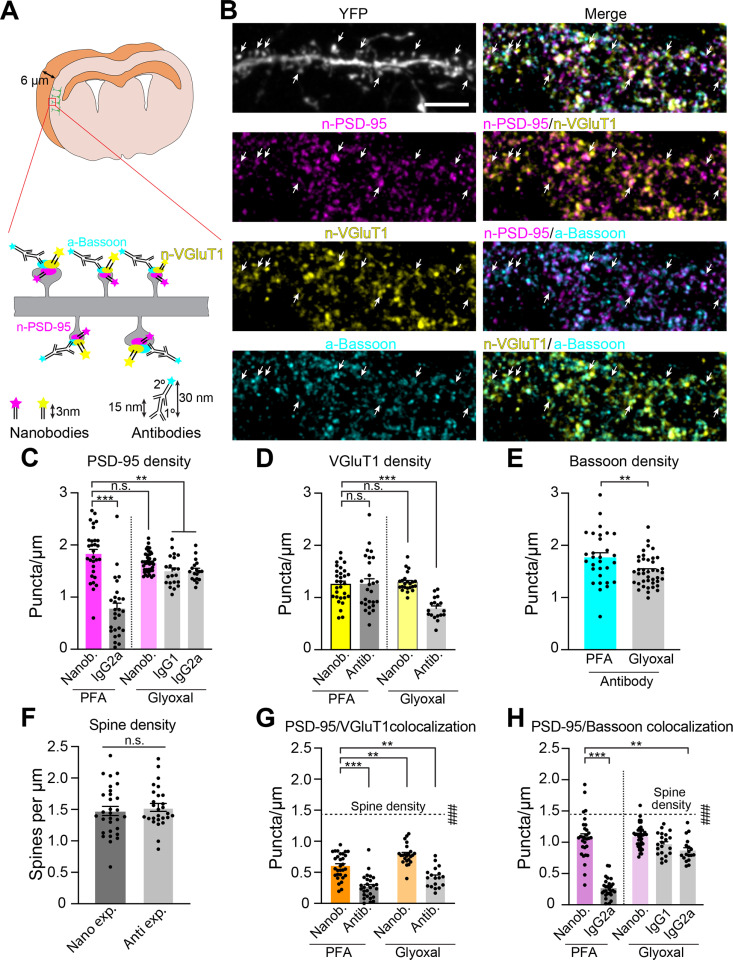
Consistent labeling of putative synapses by nanobodies. **(A)** Schematic of synaptic immunolabeling. **(B)** Representative four-channel confocal images of L5 apical dendrite in 6 µm cryosections from Thy1-YFP-H mice. PSD-95 (magenta) and VGluT1 (yellow) were visualized with nanobodies conjugated to Abberior STAR 635P and Abberior STAR 542, respectively. Bassoon (cyan) was labeled using antibodies. Arrows indicate dendritic spines. Scale bar, 5 µm. **(C–E)** Average cluster densities along YFP-labeled dendrites in PFA and glyoxal (see S4 Fig) compared to nanobody in PFA. **(C)** PSD-95 (PFA, nanobody: *n* =  30 neurons; IgG2A from S1 Fig NT condition: *n* =  27 neurons) and glyoxal (nanobody: *n* =  42 neurons; IgG1: *n* =  21 neurons; IgG2A: *n* =  18 neurons, ***p < 0.0001, *p < 0.05), **(D)** VGluT1 (nanobody, PFA: *n* =  30; glyoxal: *n* =  23; antibody, PFA: *n* =  27; glyoxal: *n* =  18, ***p < 0.0001, one-way analysis of variance [ANOVA], Dunnett’s post hoc), and **(E)** Bassoon (PFA: *n* =  30 neurons; glyoxal: *n* =  41 neurons, **p =  0.0065, unpaired Student’s *t* test). **(F)** Average spine densities on YFP-labeled apical dendrites in nanobody (*n* =  30 neurons) and antibody conditions (*n* =  27 neurons [S1E Fig]; *p* =  0.5532, unpaired Student’s *t* test). **(G)** Densities of colocalized PSD-95 and VGluT1 clusters along YFP-labeled dendrites in PFA and glyoxal (**p < 0.0033, ***p < 0.0001, one-way ANOVA, Dunnett’s post hoc). Dotted line shows spine densities from **F** (^###^*p* < 0.0001, one-way ANOVA, Dunnett’s post hoc). **(H)** Densities of colocalized PSD-95 and Bassoon clusters along YFP-labeled dendrites in PFA and glyoxal (**p < 0.008, ***p < 0.0001, one-way ANOVA, Dunnett’s post hoc), Dotted line shows spine densities from **F** (^###^*p* < 0.0001, one-way ANOVA, Dunnett’s post hoc). Bar graphs represent the mean ±  SEM. Data were collected from a minimum of 10 different neurons (dots on graph) from brain sections acquired from at least two biological replicates. The source data for panels C-H can be found in S2 Table.

To more robustly identify synapses in their native environment, we explored the advantages of using single-domain nanobodies as affinity probes for immunolabeling (Fig 1A) [44,46,47]. Cryosections from Thy-1-YFP-H animals were labeled with nanobodies for PSD-95 and VGluT1 directly conjugated to Abberior STAR 635P and Atto 542 fluorophores, respectively. The PSD-95 nanobody does not recognize other MAGUK family members [46]. The VGluT1 nanobody strongly colocalizes with VGluT1 but not VGluT2 antibodies, confirming its specificity in distinguishing VGluT1 from VGluT2 inputs (S3 Fig). We co-immunostained the same cryosections with the antibody against Bassoon and determined synaptic puncta densities along the YFP-labeled apical dendrites of L5 pyramidal neurons (Fig 1B-1E).

Labeling of PSD-95 with the nanobody significantly increased PSD-95 cluster density compared to the antibody in PFA-fixed cryosections (Fig 1C). In contrast, VGluT1 puncta densities identified by nanobody and antibody labeling were similar in PFA-fixed cryosections (Fig 1D). Importantly, a greater fraction of PSD-95 colocalized with VGluT1 or Bassoon puncta, reflecting enhanced PSD-95 labeling (S2 Fig). Based on our measurements of dendritic spine densities, the mean density of colocalized PSD-95 and Bassoon puncta identified ~ 73% of putative excitatory synapses (Fig 1F, 1H). Nonetheless, the density of nanobody-labeled PSD-95 clusters that colocalized with either VGluT1 or Bassoon was still significantly lower than the density of dendritic spines (Fig 1G, 1H). Altogether, labeling with nanobodies improved the identification of putative synapses in PFA-fixed cryosections.

### PSD-95 antibody, but not nanobody, shows improved labeling in glyoxal-fixed tissue

Recently, glyoxal fixation was shown to improve antibody labeling of synaptic proteins [41]. We therefore compared the efficiency of nanobodies and antibodies in glyoxal-fixed cryosections. We focused on PSD-95, VGluT1, and SYT1 for which both antibodies and nanobodies are available (S4A, S4B Fig). Consistent with published literature, glyoxal fixation significantly improved detection of PSD-95 using two different antibodies, which resulted in enhanced colocalization of PSD-95 with Bassoon clusters (S4C, S4G Fig). Notably, in both 4% PFA and glyoxal, nanobody reliably labeled PSD-95, and PSD-95 cluster densities identified by nanobody were significantly higher than for either of the two PSD-95 antibodies (Figs 1C and S4C). VGluT1 nanobody also showed consistent labeling in both fixatives (Fig 1D). In contrast to PFA, VGluT1 antibodylabeled fewer clusters than nanobody in glyoxal (S4D Fig). We obtained similar results for SYT1 (S4E Fig). Notably, in both fixatives, the colocalization of PSD-95 with Bassoon, VGluT1, and SYT1 was more robust when nanobodies were used to label these proteins (Figs 1G, 1H and S4G-S4I).

In glyoxal, nanobody-labeled PSD-95 and SYT1 clusters appeared brighter than clusters labeled with antibodies (S4A, S4B Fig) [46,47]. Consistent with their sub-nanomolar affinities, nanobodies bound to expressed PSD-95 and SYT1 in HEK 293T cells over a broad range of concentrations, indicating that immunolabeling with nanobodies can be highly quantitative (S5 Fig). Altogether, our results suggest that staining of PSD-95, VGluT1, and SYT1 with nanobodies results in reliable, high-affinity labeling of putative synapses, in both PFA and glyoxal.

### Nanobodies enhance apparent resolution of synaptic proteins using STED microscopy

Due to their small size and direct conjugation to fluorophores, which reduces linkage error, nanobodies have the potential to enhance resolution [43,44]. To investigate this, we undertook 3D tau-STED imaging, which combines optical signals from conventional STED with physical information of the fluorescent lifetime imaging (FLIM) of individual photons [48]. We resolved GATTA bead nano-rulers separated by 50 nm without the need for deconvolution (S6A-S6C Fig). While this distance may be at the resolution limit of our STED system, we could easily discriminate between beads labeled with two different fluorophores that are 70 nm apart (S6D, S6E Fig) [24]. All of our images are collected using Z-resolved STED, which allows for the separation of two objects in the axial plane that are only 90 nm apart (S6F-S6L Fig). Because the size of PSD-95 nanoclusters (NCs) is ~100−200 nm, our calibrated tau-STED resolution is adequate for the visualization of PSD-95 organization in spine synapses [14,15,25].

**Fig 2:**
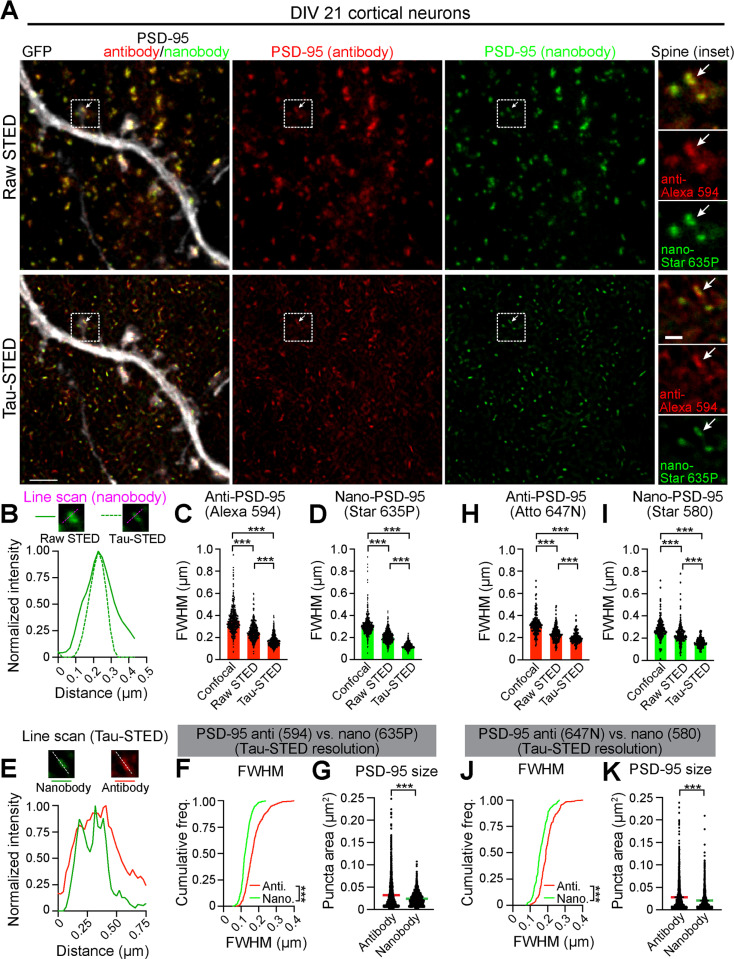
Nanobodies enhance apparent resolution of synaptic proteins using STED microscopy. **(A)** Representative raw STED and tau-STED images of a GFP-transfected dendrite (gray) from DIV21 cortical neurons co-stained with PSD-95 antibody (red, Alexa Fluor 594) and PSD-95 nanobody (green, Abberior STAR 635P). Inset shows a spine co-labeled with PSD-95 antibody and nanobody (arrow). Scale bars: 2 µm, inset: 500 nm. **(B)** Line profiles across a single PSD-95 nanocluster (NC) (arrows in panel **A**) in raw and tau-STED images. **(C)** Average FWHM of the PSD-95 antibody-labeled clusters in confocal (*n* =  429 clusters), raw STED (*n* =  444), and tau-STED (*n* =  458, ***p < 0.0001, one-way ANOVA, Tukey’s post hoc). **(D)** Average FWHM of the PSD-95 nanobody-labeled clusters in confocal (*n* =  425 clusters), raw STED (*n* =  447), and tau-STED (*n* =  469, ***p < 0.0001, one-way ANOVA, Tukey’s post hoc). **(E)** Line profiles of a tau-STED-resolved PSD-95 NC (arrows in panel **A**) co-labeled with PSD-95 antibody and nanobody. **(F)** Cumulative frequency distributions of FWHM of tau-STED-resolved PSD-95 NCs labeled by antibody (red line, *n* =  458 NCs) and nanobody (green line, *n* =  469; ***p < 0.0001, Kolmogorov–Smirnov test). **(G)** Average areas of tau-STED-resolved PSD-95 NCs labeled by antibody (red line, *n* =  2,489 NCs) and nanobody (green line, *n* =  1,519; ***p < 0.0001, unpaired Student’s *t* test). **(H)** Average FWHM of antibody-labeled PSD-95 clusters (Atto 647N) across imaging modes (confocal: *n* =  186, raw STED: *n* =  188, tau-STED: *n* =  189 clusters, ***p < 0.0001, one-way ANOVA, Tukey’s post hoc). **(I)** Average FWHM of nanobody-labeled PSD-95 clusters (Abberior STAR 580) across imaging modes (confocal: *n* =  184, raw STED: *n* =  188, tau-STED: *n* =  189 clusters, ***p < 0.0001, one-way ANOVA, Tukey’s post hoc). **(J)** Cumulative frequency distributions of FWHM of tau-STED-resolved PSD-95 NCs labeled by antibody (red line, *n* =  189 NCs) and nanobody (green line, *n* =  189 NCs; ***p < 0.0001, Kolmogorov–Smirnov test). **(K)** Average areas of tau-STED-resolved PSD-95 NCs labeled by antibody (red line, *n* =  1,744 NCs) and nanobody (green line, *n* =  1,441 NCs; ***p < 0.0001, unpaired Student’s *t* test). Bars show mean ±  SEM. Data were collected from at least three neurons across three separate transfection experiments. Dots on the bar graphs represent individual PSD-95 clusters. The source data for panels C, D, F, G, H, I, J, and K can be found in S2 Table.

We imaged PSD-95 NCs in spines of days in vitro (DIV) 21 cortical neurons transfected with green fluorescent protein (GFP) and co-immunostained with PSD-95 antibody (indirectly labeled with Alexa Fluor 594) and PSD-95 nanobody (directly conjugated to Abberior STAR 635P) (Fig 2A). As expected, raw STED significantly reduced PSD-95 cluster sizes compared to confocal imaging, with FWHM values of 255 ±  3.6 nm for antibody-labeled and 209 ±  2.5 nm for nanobody-labeled puncta, aligning with previously reported values (Fig 2C, 2D) [14,15,25]. Tau-STED further reduced PSD-95 FWHM and areas for antibody (172 ±  2.4 nm, 0.032 ±  0.0006 µm²) and nanobody (126 ±  1.3 nm, 0.023 ±  0.0003 µm²) labeled clusters, which helped improve discrimination of closely spaced NCs identified by the nanobody (Fig 2B-2G). We obtained similar results when we switched fluorophores to Abberior STAR-580 for the PSD-95 nanobody and Atto 647N for the PSD-95 antibody (Fig 2H-2K). To ensure that the resolution provided by the PSD-95 nanobody was not due to competition for binding sites between small and large probes, we imaged PSD-95 using a single probe at a time (S7A-S7C Fig). When imaged using 775 pulsed STED beam, the PSD-95 nanobody exhibited a smaller FWHM compared to the antibody. The SYT1 nanobody behaved similarly (S7D-S7F Fig). However, in both cases, these results were obtained using fluorophores of different STED efficiencies. Thus, given the small size of the two reagents, the differences in their apparent resolution should be interpreted with caution. Along these lines, the effect on resolution between nanobody and antibody was muted for VGluT1 clusters that were imaged using fluorophores compatible with the CW 660 nm STED line, which provides lower resolving power than the pulsed 775 nm line (S7G-S7I Fig). Thus, while nanobodies tend to provide increased resolution, it is highly dependent on the choice of fluorophore.

### PSD-95 nanobody reliably identifies trans*-*synaptic nanodomains in situ using STED microscopy

STED nanoscopy is well suited for probing nanoscale organization of synapses in brain tissue due to its near-infrared (775 nm) STED that attenuates light absorption and scattering [49]. Furthermore, tau-STED enables fast, simultaneous imaging of multiple fluorophores at reduced STED powers, minimizing bleaching without compromising resolution (Fig 2) [24]. We reasoned that combining tau-STED nanoscopy with nanobody labeling of cryosections might increase the accuracy of trans*-*synaptic NC (nanomodules) identification in situ.

**Fig 3:**
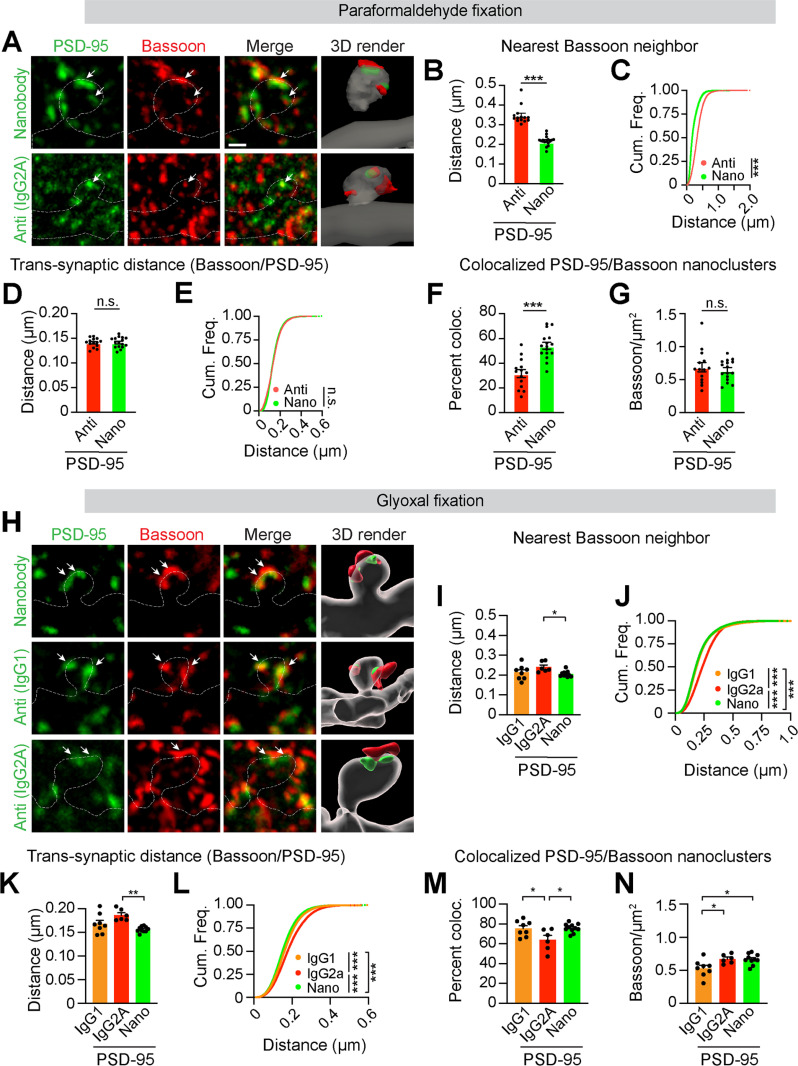
PSD-95 nanobody reliably identifies trans*-*synaptic nanodomains in situ using STED microscopy. **(A)** Two-channel tau-STED images and 3D reconstructions of PSD-95 (green) labeled by nanobody or antibody and Bassoon (red) in dendritic spines (YFP, dotted outline) in cryosections fixed in 4% PFA. Arrows indicate aligned PSD-95 and Bassoon. **(B)** Average center-to-center distances between Bassoon and the nearest PSD-95 nanoclusters (NCs) labeled with antibody (*n* =  14 images) or nanobody (*n* =  16, ***p < 0.0001, unpaired Student’s *t* test). **(C)** Cumulative distribution of nearest Bassoon – PSD-95 NC centers labeled with PSD-95 antibody (*n* =  68,806 clusters) or nanobody (*n* =  39,405 clusters, ***p < 0.0001, Kolmogorov–Smirnov test). **(D)** Average center-to-center distances between colocalized Bassoon and PSD-95 NCs labeled by PSD-95 antibody and nanobody (*p* =  0.9037, unpaired Student’s *t* test). **(E)** Cumulative frequency distributions of center-to-center distances between trans*-*synaptic Bassoon – PSD-95 NCs labeled by PSD-95 antibody (*n* =  12,992 clusters) and nanobody (*n* =  26,092, *p* = 0.4483, Kolmogorov–Smirnov test). **(F)** Percent colocalization between Bassoon and antibody- or nanobody-stained PSD-95 NCs (***p < 0.0001, unpaired Student’s *t* test). **(G)** Average Bassoon NC density per 25 × 25 µm image (*p* =  0.2518, unpaired Student’s *t* test). **(H)** Two-channel tau-STED images and 3D reconstruction of Bassoon (red) and PSD-95 (green) labeled by nanobody or two antibodies in dendritic spines (YFP, dotted outline) from cryosections fixed in glyoxal. Arrows indicate aligned PSD-95 and Bassoon. **(I)** Average center-to-center distances between Bassoon and the nearest PSD-95 NCs labeled with antibody (IgG1, *n* =  8; IgG2A, *n* =  6 images) or nanobody (*n* =  11, * *p* < 0.05, one-way ANOVA, Tukey’s post hoc). **(J)** Cumulative distribution of nearest Bassoon and PSD-95 NC centers, labeled with PSD-95 antibody (IgG1, *n* =  26,673; IgG2A, *n* =  32,533 clusters) or nanobody (*n* =  59,420, ***p < 0.0001, Kruskal–Wallis, Dunn’s post hoc). **(K)** Average trans*-*synaptic Bassoon and PSD-95 center-to-center distances (**p < 0.0005, one-way ANOVA, Tukey’s post hoc). **(L)** Cumulative frequency distributions of aligned Bassoon – PSD-95 center-to-center distances in PSD-95 antibody (IgG1, *n* =  20,656; IgG2A, *n* =  17,709 clusters) and nanobody (*n* =  43,476, ***p < 0.0001, Kruskal–Wallis, Dunn’s post hoc). **(M)** Percent colocalization between Bassoon and PSD-95 NCs (*p <  0.05, one-way ANOVA, Tukey’s post hoc). **(N)** Average Bassoon NC density (*p <  0.05, one-way ANOVA, Tukey’s post hoc). Bar graphs represent mean ±  SEM. Data were collected from a minimum of three different neurons (dots) acquired from two biological replicates. Scale bar: 500 nm applies for A, H. The source data for panels B-G and I-N can be found in S2 Table.

We labeled 6 µm Thy1-YFP-H brain cryosections with either PSD-95 nanobody or antibody and used Bassoon antibody to visualize active zones. We imaged YFP-labeled spine morphology using confocal mode, while PSD-95 and Bassoon were imaged using a 3D resolved two-color tau-STED method (Fig 3A). We segmented and analyzed X, Y, and Z-resolved Bassoon and PSD-95 NCs in an unbiased manner using the DiAna macro in ImageJ [24,50]. We 3D-rendered confocal and tau-STED images in Neurolucida 360 to aid in the visualization of PSD-95 and Bassoon in YFP-labeled spines.

We quantified the degree of PSD-95 localization near active zones by comparing the nearest-neighbor distances between the centers of Bassoon and PSD-95 NCs to the trans*-*synaptic distance of colocalized PSD-95 and Bassoon (Fig 3B-3E). Bassoon’s nearest PSD-95 neighbor was, on average, within 200 nm when we used the PSD-95 nanobody. In contrast, the nearest Bassoon neighbors were significantly further (350 nm) when PSD-95 was labeled with the antibody. One explanation for this increased distance is that the nearest Bassoon/PSD-95 neighbors are located at different synapses rather than across the synaptic cleft. We next analyzed the center-to-center distances of only those PSD-95/Bassoon NCs that overlapped at least by one pixel, which we designated as trans*-*synaptic NCs (S8A Fig). Although the trans*-*synaptic distances between the centers of Bassoon and PSD-95 were ~140 nm in both conditions, they more closely approximated the nearest-neighbor distances between Bassoon and PSD-95 nanobody-labeled NCs (Fig 3D, 3E) [24]. Indeed, labeling with the PSD-95 antibody identified fewer trans*-*synaptic NCs compared to the nanobody (Fig 3F).

Since Bassoon NC density was similar in both conditions, the large difference in nearest-neighbor distances between PSD-95 and Bassoon is likely due to the lower efficiency of PSD-95 antibody labeling in PFA-fixed brains (Fig 3G). Indeed, glyoxal fixation improved PSD-95 antibody colocalization with Bassoon, with nearest-neighbor distances more closely approximating trans*-*synaptic distances (Fig 3H-3L ). As a result, the proportion of trans*-*synaptic PSD-95/Bassoon pairs was indistinguishable from PSD-95 nanobody-labeled trans*-*synaptic nanodomains (Fig 3M, 3N). Notably, glyoxal fixation did not further improve PSD-95 nanobody labeling as indicated by the similar nearest-neighbor distances between PSD-95 and Bassoon NCs and the comparable percentage of trans*-*synaptic nanomodules in PFA-fixed brain sections. Thus, while glyoxal enhances trans*-*synaptic nanodomain detection for the PSD-95 antibody, the PSD-95 nanobody reliably labels PSD-95 opposite Bassoon NCs in both PFA- and glyoxal-fixed brains.

Because of anisotropic X, Y, and Z resolution, nanomodule orientation may potentially affect the distances between pre- and post-synaptic proteins (S8 Fig). Therefore, we determined the distances between the centers of PSD-95 and Bassoon NCs aligned in either the XY or Z plane and found no significant differences (S8B Fig). Thus, center-to-center distances allow reliable identification of aligned NCs regardless of their orientation. Moreover, Z-projected clusters and spines were less abundant, potentially minimizing the impact of anisotropy in our data set (S8C-S8E Fig). Altogether, our tau-STED imaging of brain cryosections enables simultaneous visualization of multiple endogenous synaptic molecules to assign their nano-organization to spines of varying morphologies with high reliability.

### TC synapses in layer 5 of S1 are larger than neighboring CC synapses despite similar trans*-*synaptic nano-organization

Using our tau-STED imaging approach in thin cryosections, we next examined how different types of excitatory synapses in the brain organize their nano-architecture. L5 pyramidal neurons are a great model for examining this question due to their vast dendritic trees spanning layers 1–5. In S1, they receive glutamatergic input from CC and TC afferents in both apical and basal dendritic domains that have been functionally characterized in vivo [31,51]. In the brain, CC and TC synapses can be distinguished by the presence of either VGluT1 or VGluT2, respectively, allowing for the direct comparison of these glutamatergic synaptic subtypes in the same cell [52,53].

**Fig 4:**
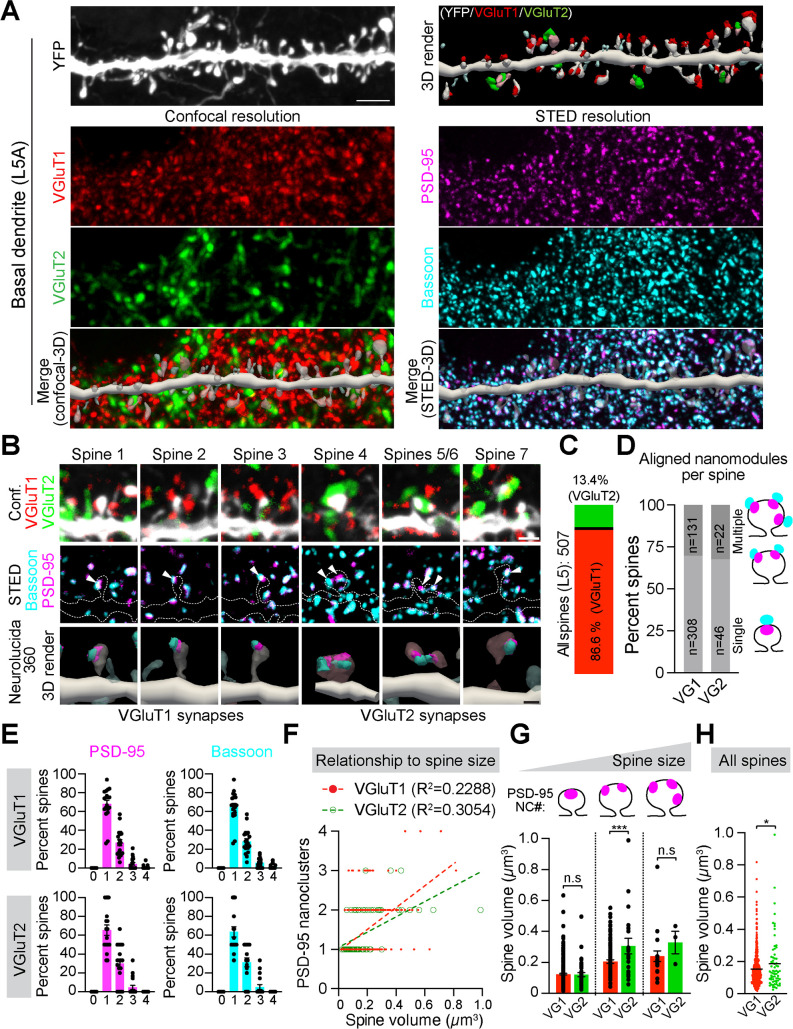
TC synapses in layer 5 of **S1** **are larger than neighboring CC synapses despite similar trans*-*synaptic nano-organization.** **(A)** Five-channel confocal/tau-STED images of YFP-labeled dendritic spines on basal dendrites of L5 pyramidal neurons in S1 of Thy-1-YFP-H mice. YFP (gray), VGluT1 (red), and VGluT2 (green) were imaged in confocal (left), while PSD-95 (magenta) and Bassoon (cyan) were imaged in STED (right). 3D reconstruction with Neurolucida 360 to classify VGluT1^ +^ or VGluT2^ +^ spines and localize PSD-95 and Bassoon nanoclusters (NCs). Scale bar, 2 µm. **(B)** Sub-stacks of individual dendritic spines receiving either VGluT1 (red) or VGluT2 (green) input (top row). Tau-STED-resolved PSD-95 (magenta) and Bassoon (cyan) NCs (arrows) in the same spines (middle row, dotted outlines). 3D-reconstructed spines with corresponding PSD-95 and Bassoon NCs (bottom row). High-contrast images are shown. Scale bars, 1 µm (tau-STED), 500 nm (3D render). **(C)** Fraction of TC and CC synapses identified by VGluT1 and VGluT2 staining of 507 spines. **(D)** Fraction of VGluT1^ +^ (*n* =  439) and VGluT2^ +^ (*n* =  68) spines with single or multiple aligned PSD-95 and Bassoon NCs. **(E)** Distribution of VGluT1^ +^ and VGluT2^ +^ spines based on the number of PSD-95 and Bassoon. **(F)** Linear correlation between spine size and PSD-95 NC numbers in VGluT1^ +^ (*R*^2^ =  0.2288, slope =  2.781 ±  0.48) and VGluT2^ +^ (*R*^2^ =  0.3054, slope =  1.942 ±  0.72) spines (*p* =  0.0711, ANCOVA). **(G)** Comparison of spine size in VGluT1^ +^ and VGluT2^ +^ spines with one (VG1: *n* =  308, VG2: *n* =  44, *p* =  0.3484), two (VG1: *n* =  107, VG2: *n* =  21, ***p =  0.0008), and three PSD-95 NCs (VG1: *n* =  20, VG2: *n* =  3, *p* =  0.3582, unpaired Student’s *t* test). **(H)** Average sizes of all VGluT1^ +^ and VGluT2^ +^ spines regardless of the number of NCs (*p =  0.0263, unpaired Student’s *t* test). Bars represent the mean ±  SEM acquired from spines (dots, panels G, H) of 19 different basal dendrites (dots panel E) of L5 neurons obtained from two biological replicates. The source data for panels E-H can be found in S2 Table.

We initially focused on the nano-organization of TC synapses on basal dendrites (L5) where afferents from the posterior-medial (POm) thalamic nucleus generate robust post-synaptic responses [51]. We collected 6 µm brain sections from S1 of Thy1-YFP-H mice and immunolabeled them with nanobodies against VGluT1 and PSD-95, and antibodies against VGluT2 and Bassoon. We then performed simultaneous five-channel confocal and STED imaging (Fig 4A). YFP, VGluT1, and VGluT2 were imaged in confocal resolution to distinguish between dendritic spines receiving TC (VGluT2^+^) and CC (VGluT1^+^) input (Fig 4B, top row). PSD-95 and Bassoon were imaged in Z-resolved (3D) tau-STED to determine the trans*-*synaptic nano-organization in VGluT1^ +^ and VGluT2^ +^ spines (Fig 4B, middle row). We acquired ~2 µm stacks through basal dendrites and reconstructed confocal and STED images in Neurolucida 360 to create a 3D rendering of dendritic spines and synaptic clusters (Fig 4A, 4B). Only those synaptic clusters that colocalized with YFP-labeled spines were analyzed (Fig 4A, top right).

We imaged a total of 507 dendritic spines on basal dendrites. 3D reconstructions revealed that only 68 (13.4%) spines were TC synapses based on VGluT2 colocalization. The remaining 439 spines (86.6%) were identified as CC synapses by their VGluT1 labeling (Fig 4C). We next determined PSD-95 and Bassoon nano-organization in each spine subtype (Fig 4D). Discrete, aligned PSD-95 and Bassoon NCs were identified as nanomodules (S8 Fig) [24,25]. The majority (70%) of VGluT1^ +^ spines had a single nanomodule, while 30% contained multiple (two, three, or four) nanomodules. (Fig 4B, 4D). Similarly, 68% of VGluT2^ +^ spines contained a single aligned PSD-95 and Bassoon, while 32% had multiple (Fig 4B, 4D). Thus, the highest proportion of spines in both categories contained only one PSD-95 and Bassoon NC, while spines with two, three, and four NCs were less common (Fig 4E).

Previous studies demonstrated a linear relationship between the number of PSD-95 NCs and spine size [24,25]. In agreement with this rule, the number of PSD-95 NCs scaled with the size of both VGluT1^ +^ and VGluT2^ +^ spines (Fig 4F). A limitation of this analysis is that it cannot predict nanomodule numbers from spine volume. Indeed, despite a similar linear relationship, VGluT2^ +^ spines were larger on average than VGluT1^ +^ spines (Fig 4H), as shown previously [52,54,55]. To determine the nanoscale logic for the overall larger size of spines receiving TC input, we compared sizes of VGluT1^ +^ and VGluT2^ +^ spines with one, two, and three PSD-95 NCs (Fig 4G). VGluT1^ +^ and VGluT2^ +^ spines containing only one PSD-95 had similar sizes. However, VGluT2^ +^ spines containing two PSD-95 NCs were significantly larger than VGluT1^ +^ spines containing the same number of PSD-95 NCs (Fig 4G). We observed a similar trend for spines with three PSD-95 NCs, but the difference was not statistically significant (*p* =  0.2408, *t* test), likely because there were only three VGluT2^ +^ spines with three NCs. Thus, unless individual PSD-95 nanodomains in VGluT2^ +^ spines occupy a significantly smaller portion of the post-synaptic membrane than in VGluT1^ +^ spines, it is possible that the NCs themselves are larger, challenging the rules of modularity [25].

### VGluT2^ +^ spines with multiple PSD-95/Bassoon nanomodules are abundant on apical dendrites of L5 pyramidal cells

**Fig 5:**
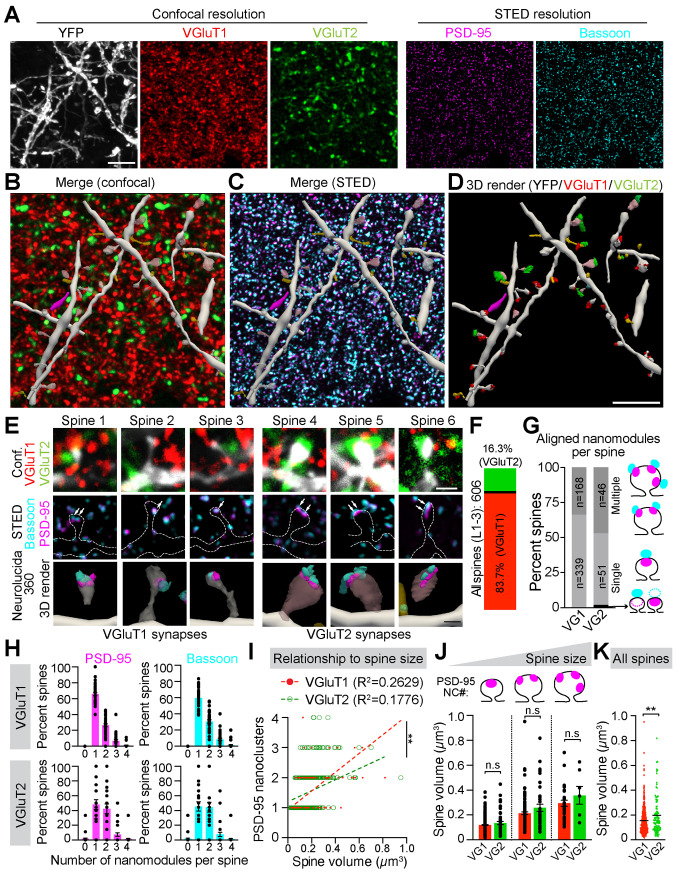
VGluT2 ^+^ spines with multiple PSD-95/Bassoon nanomodules are abundant on apical dendrites of L5 pyramidal cells. **(A)** Five-channel confocal/tau-STED images of YFP-labeled dendritic spines on apical dendrites of L5 pyramidal neurons in S1 of Thy-1-YFP-H mice. YFP (white), VGluT1 (red), and VGluT2 (green) were imaged in confocal mode (left), while PSD-95 (magenta) and Bassoon (cyan) were imaged in STED mode (right). **(B-D)** 3D reconstruction with Neurolucida 360 to classify VGluT1^ +^ or VGluT2^ +^ spines and localize PSD-95 and Bassoon nanoclusters (NCs). Scale bar (A-D), 5 µm. **(E)** Sub-stacks of individual dendritic spines receiving either VGluT1 (red) or VGluT2 (green) input (top row). Tau-STED-resolved PSD-95 (magenta) and Bassoon (cyan) NCs (arrows) in the same spines (middle row, dotted outlines). 3D-reconstructed spines with corresponding PSD-95 and Bassoon NCs (bottom row). High-contrast images are shown. Scale bars, 1 µm (tau-STED), 500 nm (3D render). **(F)** Quantification of the fraction of TC and CC synapses determined by VGluT1 and VGluT2 staining of 606 spines. **(G)** Quantification of the fraction of VGluT1^ +^ (*n* =  507) and VGluT2^ +^ (*n* =  99) spines with single or multiple aligned PSD-95 and Bassoon nanomodules. **(H)** Distributions VGluT1^ +^ or VGluT2^ +^ spines with the indicated numbers of PSD-95 or Bassoon NCs. **(I)** Linear correlation between spine size and PSD-95 NC numbers in VGluT1^ +^ (*R*^2^ =  0.2629, slope =  3.122 ±  0.464) and VGluT2^ +^ (*R*^2^ =  0.1776, slope =  1.833 ±  0.799) spines (**p =  0.0037, ANCOVA). **(J)** Comparison of spine size in VGluT1^ +^ and VGluT2^ +^ spines with one (VG1: *n* =  329 spines, VG2: *n* =  49 spines, *p* =  0.5722), two (VG1: *n* =  133 spines, VG2: *n* =  39 spines, *p* =  0.0931) and three PSD-95 NCs (VG1: *n* =  31 spines, VG2: *n* =  8 spines, *p* =  0.1368, unpaired Student’s *t* test). **(K)** Average sizes of all VGluT1^ +^ and VGluT2^ +^ spines regardless of the number of NCs (**p =  0.0014, unpaired Student’s *t* test). Bars represent the mean ±  SEM acquired from spines (dots in J, K) on 37 apical dendritic segments (dots in H) of L5 neurons from two biological replicates. The source data for panels H-K can be found in S2 Table.

Layer 5 pyramidal cells also receive the POm TC input in their apical domains (L1-3) [31,51]. Therefore, we subjected apical dendrites of L5 pyramidal neurons to five-channel confocal and tau-STED imaging of TC and CC synapses (Fig 5A-5E and S1-S3 Videos).

Of the 606 dendritic spines we imaged on apical dendrites, only 16.3% were TC synapses, while 83.7% were classified as CC synapses (Fig 5F). The nano-organization of VGluT1^ +^ spines in L1-3 was similar to spines in L5, with most spines displaying a single aligned PSD-95/Bassoon nanomodule. In contrast, fewer VGluT2^ +^ spines (51%) contained a single aligned PSD-95/Bassoon, while 46% had multiple nanomodules (Fig 5G). Thus, the distribution of PSD-95 and Bassoon NCs in VGluT2^ +^ spines shifted relative to VGluT1^ + ^spines, resulting in nearly equal proportions of spines with one or two NCs (Fig 5H). VGluT2^ +^ and VGluT1^ +^ spines with three and four NCs were uncommon (<6%). It is important to note that we saw only one or two VGluT2^ +^ spines with any given nano-organization per dendritic segment due to their low abundance in the cortex. Thus, even though proportionally there were more VGluT2^ +^ spines with multiple aligned PSD-95/Bassoon nanomodules in L1-3, VGluT1^ +^ spines with two or more nanomodules were still more abundant on apical dendrites of L5 cells.

Given the differences in the pattern of PSD-95 and Bassoon nano-organization in VGluT1^ +^ and VGluT2^ +^ spines, we wondered whether the relationship between nanomodule numbers and spine size might also differ. Both VGluT1^ +^ and VGluT2^ +^ spine sizes showed a linear correlation with PSD-95 NC numbers (Fig 5I). However, the slope of this linear relationship was significantly lower for VGluT2^ +^ spines compared to VGluT1^ +^ spines (*p* =  0.0037, ANCOVA). We reasoned that this is due to fewer VGluT2^ +^ spines with one PSD-95 NC and more VGluT2^ +^ spines with two PSD-95 NCs compared to VGluT1^ +^ synapses. To shed light on this skewed relationship, we compared sizes of VGluT1^ +^ and VGluT2^ +^ spines with one, two, and three PSD-95 NCs (Fig 5J). We found no significant differences in the average volumes of VGluT1^ +^ and VGluT2^ +^ spines within each category, indicating that apical VGluT1^ +^ and VGluT2^ +^ spines with equivalent numbers of PSD-95 NCs are similar with respect to their size. Yet, when comparing all spines in L1-3 regardless of their PSD-95 NC numbers, VGluT2^ +^ spines were significantly larger on average than VGluT1^ +^ spines (Fig 5K). Thus, CC and TC synapses on the apical dendrites of L5 neurons exhibit comparable organizational principles. However, a key distinction is the greater prevalence of VGluT2^ +^ spines with multiple nanomodules, which are larger.

### Proteome expansion confirms distinct populations of L1/3 dendritic spines with discrete VGluT1 and VGluT2 clusters

We next undertook deep structured illumination microscopy (SIM) of Thy-1-YFP-H brain sections in which we expanded the proteome to verify the accurate assignment of clusters to individual YFP-labeled spines and to define their relationship to pre-synaptic input [56]. Following the expansion of brain sections, we labeled them with VGluT1 and VGluT2 antibodies along with appropriate secondary antibodies to visualize the vesicle clusters on individual spines (S9A Fig). Using 3D projections, we verified that VGluT1 and VGluT2 contacted specific YFP-labeled dendritic spines (S9B, S9C Fig, and S4 Video). Consistent with our confocal imaging, 73% of spines received VGluT1 input, while only 19% were contacted by VGluT2 (S9E Fig). Of the 364 spines, only seven were contacted by both VGluT1 and VGluT2, further supporting the accurate attribution of nanodomains to specific spines. Importantly, both VGluT1 and VGluT2 formed discrete clusters on the surface of YFP-labeled spines (S9D Fig). Similar to our STED analysis of aligned PSD-95/Bassoon nanomodules, most VGluT1^ +^ spines (~69%) contained a single VGluT1 cluster. In contrast, nearly half of VGluT2^ +^ spines (45%) had multiple (two and three) vesicle clusters (S9F Fig). These results complement our findings that many VGluT2^ +^ spines on apical dendrites of L5 neurons form synapses with multiple nanodomains and suggest that these synaptic sites also contain multiple vesicular clusters.

### TC synapses in apical and basal compartments of L5 neurons are built on distinct nanoscale principles

TC synapses on both basal and apical dendrites of L5 pyramidal neurons are larger than CC synapses. While the larger average size of apical VGluT2^ +^ spines is due to the higher relative abundance of spines containing multiple nanomodules, the same logic cannot be applied to VGluT2^ +^ spines on basal dendrites. Indeed, most VGluT2^ +^ spines in L5 contain a single aligned PSD-95/Bassoon NC. Thus, TC synapses in apical and basal domains may adhere to distinct organizational principles.

**Fig 6:**
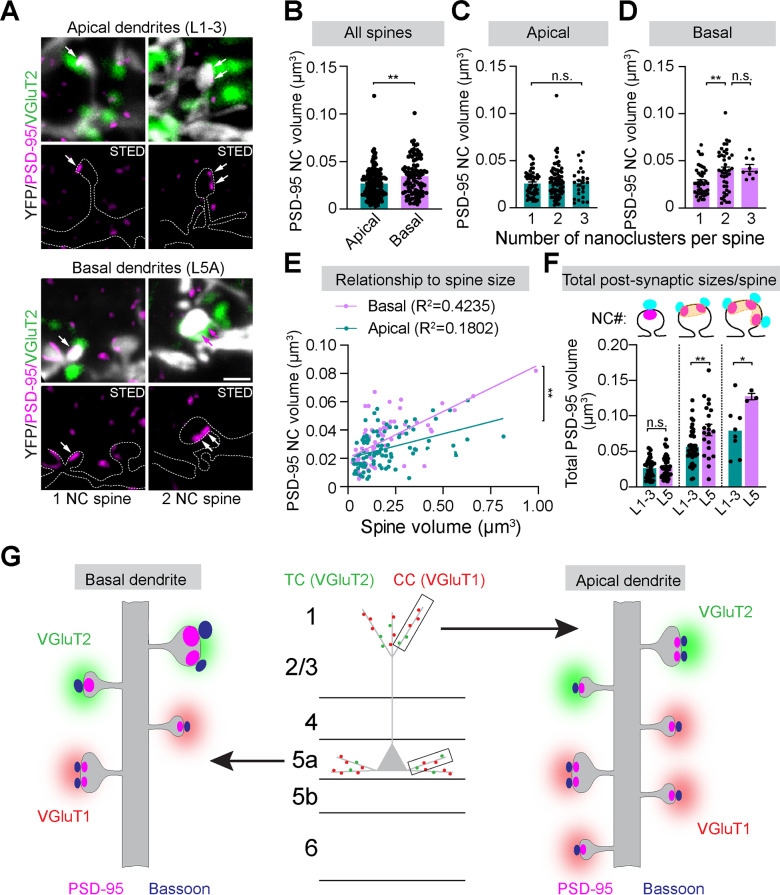
TC synapses in apical and basal compartments of L5 neurons are built on distinct nanoscale principles. **(A)** Representative images of single and two PSD-95 nanocluster (NC) (magenta, arrows) VGluT2^ +^ (green) YFP-labeled spines (gray, dotted outlines) from apical and basal dendrites of L5 pyramidal neurons in S1 of Thy-1-YFP-H mice. Scale bar, 1 µm. **(B)** Average PSD-95 NC volumes in all VGluT2^ +^ spines from apical (*n* =  152 NCs) and basal (*n* =  95 NCs) dendrites (**p =  0.0011, unpaired Student’s *t* test). **(C)** Average PSD-95 NC volumes in VGluT2^ +^ spines with one (*n* =  52), two (*n* =  76), and three (*n* =  24) PSD-95 on apical dendrites (*p* =  0.6376, one-way ANOVA, Tukey’s post hoc). **(D)** Average PSD-95 NC volumes in VGluT2^ +^ spines with one (*n* =  44), two (*n* =  42), and three (*n* =  9) PSD-95 on basal dendrites (**p =  0.0068 – one vs. two; *p* =  0.0774 – one vs. three; *p* =  0.9335 – two vs. three, one-way ANOVA, Tukey’s post hoc). **(E)** Relationship between the average PSD-95 NC volume per spine and VGluT2^ +^ spine size in apical (*R*^2^ =  0.1802, slope =  0.0343, 95% confidence: 0.0193−0.049, *n* =  96 spines) and basal (*R*^2^ =  0.4235, slope =  0.0658, 95% confidence: 0.0469−0.0846, *n* =  68 spines) dendrites (**p =  0.009, ANCOVA). **(F)** Average total PSD-95 volume (sum of all PSD-95 NCs per spine) in apical and basal VGluT2^ +^ spines with one (L1-3: *n* =  49 spines, L5: *n* =  44 spines, *p* =  0.5569), two (L1-3: *n* =  39 spines, L5: *n* =  21 spines, **p =  0.0047), and three (L1-3: *n* =  8 spines, L5: *n* =  3 spines, *p =  0.0476, unpaired Student’s *t* test) PSD-95 NCs. **(G)** Model of trans*-*synaptic PSD-95/Bassoon nano-organization in VGluT1^ +^ and VGluT2^ +^ spines in basal and apical compartments of L5 neurons. Bars represent the mean ±  SEM acquired from spines on 37 apical and 19 basal dendritic segments of L5 neurons from two biological replicates. Dots on graph indicate individual PSD-95 NCs. The source data for panels B-F can be found in S2 Table.

We began by analyzing sizes of individual PSD-95 NCs in VGluT2^ +^ spines from L5 and L1-3 (Fig 6). Despite similar spine volumes, VGluT2^ +^ spines in L5 had larger PSD-95 NCs than L1-3 VGluT2^ +^ spines, suggesting that two populations use NCs of different sizes to construct their nano-architecture (Fig 6A, 6B). Consistent with the modular principles of nano-organization, PSD-95 NCs in VGluT2^ +^ spines from L1-3 had similar volumes regardless of the number of NCs present (Fig 6C) [25]. In contrast, L5 VGluT2^ +^ spines with one PSD-95 had significantly smaller NCs than those with two PSD-95 (Fig 6D). Interestingly, sizes of discrete PSD-95 NCs in VGluT2^ +^ spines with two and three PSD-95 were similar (Fig 6D). This effect may reflect capping of individual NC sizes in spines with multiple (two or more) PSD-95, although it is challenging to assess from current data due to a very low number (*n* =  3) of basal VGluT2^ +^ spines with three PSD-95 nanodomains. Since NC sizes vary between spines containing different numbers of PSD-95, these findings suggest that VGluT2^ +^ spines in L5 may deviate from modular principles of nano-organization.

To better understand how closely the two spine populations adhere to rules of modularity, we correlated average sizes of PSD-95 NCs per spine as a function of increasing spine volume, regardless of NC number (Fig 6E). We reasoned that a low correlation between spine size and NC size indicates unitary NCs, consistent with modular nano-organization. In contrast, a high positive correlation would suggest that NC size increases with spine volume, deviating from modularity. We observed a significantly higher correlation between the average size of PSD-95 NCs and volumes of VGluT2^ +^ spines in L5 compared to L1-3 (*p* =  0.009, ANCOVA). Nonetheless, there was still a positive correlation between sizes of PSD-95 NCs and VGluT2^ +^ spines in L1-3, suggesting that PSD-95 NCs in these spines also increase in size with increasing spine volume. However, sizes of PSD-95 NCs in apical VGluT2^ +^ spines exhibit a constrained upper limit, suggesting regulation of their growth across spine sizes and nanomodule numbers. Such constrained size of PSD-95 NCs resembles the modular nano-organization of VGluT1^ +^ synapses [24,25].

Sizes of spines and PSDs are tightly linked, with larger spines and PSDs indicating stronger synapses [57,58]. Given the differences in PSD-95 size and organization between apical and basal VGluT2^ +^ spines, we asked how the total post-synaptic size changes with PSD-95 NC number. The total post-synaptic size was calculated by summing the volumes of all PSD-95 NCs per spine (Fig 6F). As expected, total post-synaptic sizes in VGluT2^ +^ spines in both layers increased with the number of PSD-95 NCs (Fig 6F). This effect was more robust in VGluT2^ +^ spines on basal dendrites, as L5 spines with two PSD-95 NCs had significantly larger total PSD-95 sizes than L1-3 spines with the same number of NCs (*p* =  0.0017, *t* test). Similarly, L5 VGluT2^ +^ spines with three PSD-95 NCs had significantly larger total PSD-95 sizes than their L1-3 counterparts (Fig 6F, *p* =  0.0476, *t* test). These data indicate that VGluT2^ +^ spines in L5 assemble post-synaptic macrostructures from larger nanoscale building blocks and, thus, exhibit structural correlates of stronger synapses than VGluT2^ +^ spines L1-3 [57,58].

## Discussion

A fundamental challenge for neuroscience is deciphering how rich molecular and structural diversity of active zones and PSDs endow synapses with specific functions that underlie information storage and processing in neural circuits. However, this has remained elusive due to difficulties in obtaining 3D nanoscale reconstructions of individual synapses within complex brain tissue. Using nanobodies and 3D tau-STED imaging of TC and CC synapses on L5 pyramidal neurons in the S1 cortex, we reveal a level of variability in how modular principles are applied to these two glutamatergic synapses (Fig 6G). These results shed light on the importance of the type (VGluT1 versus VGluT2) and location (apical versus basal) of afferent input in shaping synaptic nano-architecture in the brain.

Accurate reconstruction of synaptic nano-architecture in dense neuropil remains a challenge. Traditional immunolabeling in paraformaldehyde (PFA)-fixed samples often suffers from poor antibody accessibility, leading to incomplete visualization of synaptic proteins [40–42]. While techniques like non-canonical amino acid labeling and CRISPR-based fluorescent tagging of endogenous proteins circumvent some of these issues, they may lack the flexibility for simultaneous, multi-protein imaging needed to capture the molecular diversity of synapses [59–61]. Nanobodies exhibit consistent, high-affinity labeling of proteins like PSD-95 in PFA-fixed tissue. However, the limited repertoire of available nanobodies targeting endogenous synaptic proteins restricts their broader application on the level that is possible with antibodies [46,47]. Recent advancements in recombinant antibody technologies could help expand the toolkit for synaptic imaging by accelerating the development of single-chain variable domains (scFvs) that are optimized for high-resolution imaging in the brain [62–64]. Glyoxal fixation also appears promising, as it enhances labeling of core PSD proteins, PSD-95, AMPARs, and NMDARs with antibodies [41]. In our hands, fixation with glyoxal increased colocalization of PSD-95 with VGluT1 and Bassoon and significantly improved trans*-*synaptic nanodomain visualization in STED. The development of strategies that integrate nanobodies and antibodies may provide a more comprehensive approach, potentially uncovering new dimensions of synaptic diversity at single protein resolution [45].

Much of our understanding of synaptic nano-organization has come from studies on cultured neurons, typically examining a single synapse type [13–17,19,24,25]. At spine synapses, modular principles offer an insight into the relationship between pre- and post-synaptic nano-organization and its role in structural plasticity [24,25]. We show that this modular rule is flexible when comparing VGluT1^ +^ and VGluT2^ +^ spines in the brain across different cortical layers. It remains to be seen how AMPAR and NMDAR nanodomains are arranged in vivo among different synaptic types, especially given their modular yet non-overlapping organization in vitro [13,24,65]. Moreover, MAGUK family members SAP-102 and PSD-95, known to influence AMPAR and NMDAR trafficking, show differential expression in VGluT1^ +^ and VGluT2^ +^ synapses, hinting that synaptic nano-organization might not only vary by input but also by receptor subtype [66,67]. Understanding these distinctions could reveal how synapses fine-tune their molecular architecture to meet the functional requirements of diverse circuits.

It has long been proposed that TC inputs to L4-6 function as glutamatergic drivers, whereas TC inputs to L2-3 are glutamatergic modulators [68,69]. Although we could not definitively distinguish L5A and L5B TC inputs, VGluT2^ +^ spines on basal dendrites displayed larger PSD-95 NCs than TC synapses in L1-3, which aligns with evidence showing stronger TC synaptic currents on basal dendrites of L5 neurons [51]. Recent demonstration that VGluT2 input and associated PSD-95 enlarge selectively in L5 during learning suggests that these synapses play a key role in regulating experience-dependent plasticity [70]. It will be essential to determine whether TC synapses on basal dendrites lack mGluR nanodomains, a characteristic feature of glutamatergic drivers [69].

In contrast to VGluT2^ +^ spines on basal dendrites, many VGluT2^ +^ spines in L1-3 contain multiple aligned PSD-95/Bassoon nanomodules, and single spines are contacted by multiple VGluT2 NCs. While the convergence of pre-synaptic input has been proposed as a mechanism to enhance thalamic efficacy, our findings suggest the possibility of input convergence at the level of a single spine in these particular synapses [35–37]. Alternatively, these spines may fall into a category of perforated synapses with large pre-synaptic terminals [57]. However, it remains unclear whether the VGluT2 clusters originate from the same terminal or multiple distinct pre-synaptic cells, especially since existing EM data lack direct correlations with known protein distributions at synapses. Fully understanding this will require light-EM correlative imaging as well as functional studies at a single synapse level, to assess whether discrete VGluT2 NCs are linked or independent.

The structure-function relationship of TC synapses remains enigmatic. Despite their lower abundance compared to CC synapses, TC synapses are better at activating cortical neurons. The reason for this is not entirely understood, despite many studies directly comparing the function of TC and CC synapses [33,34,38,54]. Our super-resolution approach reveals heterogeneity of TC synapse nano-organization across laminae. However, TC input is not uniformly distributed across L5 and L2/3 cells but instead targets specific dendrites [28,31,38]. Thus, within the same neuron TC and CC information might be processed in distinct dendritic domains, each with specialized synapses designated to process different types of information for enhanced efficiency [38]. This idea is supported by recent work demonstrating that the nanoscopic landscape of key regulators of synaptic specification that bind Neurexins- Neuroligins and LRRTMs - differs across TC and CC inputs and cortical layers [71]. Future studies will need to determine how input/target selectivity might engage different molecular mechanisms that may establish unique subcellular and nanoscale niches for cortical processing.

## Materials and methods

### Animals

All animal studies were approved by the Institutional Animal Care and Use Committee guidelines at West Virginia University (#2102040142_R1 and #2105042129) in accordance with the US National Institutes of Health guidelines. Thy1-YFP-H mice (B6.Cg-Tg(Thy1-YFP)16Jrs/J, Strain # 003782, RRID: IMSR_JAX:003782) were obtained from Jackson Laboratory and housed (2–5 mice per cage) in West Virginia University Health Sciences Center’s laboratory animal facility. Both male and female mice were used for experiments at 1–3 months of age. Timed pregnant Long-Evans rat females used for the collection of E17-18 male and female rat embryos to make primary cortical neuron cultures were purchased from Charles River Laboratories (Wilmington, MA).

### Brain tissue collection and processing

To procure mouse brain sections, mice were perfused using previously described methods [25]. Mice were anesthetized with 4% isoflurane until unable to right themselves and first perfused transcardially at a rate of 7 mL/min for 2 min with ice-cold ACSF (125 mM NaCl, 2.5 mM KCl, 2 mM CaCl_2_, 25 mM NaHCO_3_, 1.25 mM NaH_2_PO_4_, 2 mM MgSO_4_, 10 mM Glucose, and 0.052 mg/mL Heparin, pH 7.4), followed by 60 mL ice-cold 4% PFA (ref # 00380-1, Polysciences, Warrington, PA) in 0.1 M phosphate buffer (PB) (80 mM Na_2_HPO_4_ and 19 mM NaH_2_PO_4_, pH 7.4) containing 0.003% glutaraldehyde (ref # 1875, VWR, Radnor, PA). Brains were removed and post-fixed overnight at 4 °C in 4% PFA. After washing three times (10 min each wash) in 1× PBS (ref # 70011-044, Gibco, Jenks, OK), brains were transferred to 15% sucrose solution and allowed to equilibrate overnight at 4 °C, then transferred to 30% sucrose solution and allowed to equilibrate at 4 °C for 48 h. In preparation for cryosectioning, brains were embedded in Tissue-Tek O.C.T. Compound (ref # 4583, Sakura Finetek) by submerging it in 2-Methylbutane (ref # O3551-4, Fisher Scientific, Pittsburgh, PA) cooled with dry ice to −80 °C. Brains were sectioned at 6 μm on a Leica CM3050 S cryostat set to −20 °C for both the object and chamber temperature (Leica Biosystems, Nussloch, Germany). We collected sections from the primary somatosensory cortex (coronal sections from bregma: A/P −1.05 mm to −2.1 mm). Sections were mounted on pre-treated #1.5 coverslips coated with 5 mg/mL gelatin from bovine skin and 1 mM chromium (III) potassium sulfate to ensure brain tissue was positioned near the 100×, 1.4 N/A objective to minimize surface irregularities that could compromise resolution [72]. The cryosections were then immediately used for immunohistochemistry (IHC).

As an alternative fixation method, we perfused mice with glyoxal using a modified protocol from Konno and colleagues [41]. The glyoxal solution was prepared by mixing 9% glyoxal (ref #128465, Sigma-Aldrich, Darmstadt, Germany) and 8% acetic acid (ref #A38S, Fisher Scientific) in 0.1 M PB, pH 4.0. Mice were first transcardially perfused with ice-cold ACSF (as described above), followed by 60 mL of the ice-cold glyoxal solution. Brains were then removed and post-fixed overnight at 4 °C in the glyoxal solution. We then followed the protocol for PFA perfusions to prepare the tissue and perform immunostaining.

### Immunohistochemistry of fixed cryosections

Brain sections underwent one of the following IHC protocols.

#### No treatment.

Brain sections perfused with either PFA or glyoxal were blocked and permeabilized for 1 h in 1× PBS containing 5% normal goat serum (ref # 16210064, Thermo Fisher Scientific) and 0.5% Triton X-100 (ref # BP151, Thermo Fisher Scientific). Tissue was incubated in primary antibodies or nanobodies (see S1 Table for details) diluted in blocking buffer for 48 h at 4 °C. Tissue was washed three times with 1× PBS (10 min each wash) and then incubated in secondary antibodies (S1 Table) for 2 h at room temperature. After washing three times with 1× PBS, brain sections were mounted in ProLong Glass Antifade mounting medium (ref # P36984, Invitrogen, Waltham MA) and kept at room temperature until imaging.

#### Antigen retrieval.

Before the IHC, PFA-perfused brain sections were first subjected to a 5 min incubation in MiliQ water with gentle rocking at 37 °C, followed by 2 min rocking at 37 °C in pepsin reagent (ref # R2283, Millipore-Sigma, Burlington, MA). The pepsin reaction was quenched by incubations in blocking buffer. The tissue was then subjected to immunostaining with primary and secondary antibodies as described above for the no-treatment protocol.

#### Clear unobstructed brain imaging cocktails (CUBIC).

CUBIC clearing of PFA-fixed brain sections was performed as previously described [25,73]. Brain sections were blocked overnight in blocking buffer, followed by a four-day incubation at 4 °C in primary antibodies diluted in blocking buffer. Sections were washed with 1× PBS (three times for 10 min) and then incubated overnight in secondary antibodies diluted in blocking buffer. After washing 3× in 0.1M PB, tissue was placed in the CUBIC reagent, consisting of 25% w/v urea (ref # U5378, Millipore-Sigma, St. Louis, MO), 25% w/v Quadrol (ref # 122262, Millipore-Sigma), and 0.2% w/v Triton X-100 (ref # T8787, Millipore-Sigma). Upon visible clearing, tissue was mounted in the CUBIC reagent, and slides were sealed with nail polish and imaged within 48 h.

#### CUBIC^+^  antigen retrieval.

The tissue first underwent the antigen retrieval protocol using pepsin reagent as described above, followed by immunostaining and tissue clearing described for the CUBIC protocol. Sections were mounted in the CUBIC reagent, and slides were sealed with nail polish and imaged within 48 h.

### Primary cortical neuron culture preparation

Dissociated cortical neurons were prepared from the embryonic day 17–18 (E17-18) male and female rat cerebral cortices as described previously [24,25]. Primary cortical neurons were cultured for 21–25 DIV in Neurobasal medium with phenol red (cat#: 12348017, Thermo Fisher Scientific) supplemented with 1× B27 supplement (ref # 17504044, Thermo Fisher Scientific), 200 mM L-glutamine (cat#: 25030081, Thermo Fisher Scientific) and 0.1 mg/mL penicillin-streptomycin (ref # 15140122, Thermo Fisher Scientific). Neurons were plated on poly-D-lysine (ref # 354210, Corning, Corning, NY) and laminin (mouse, ref # 354232, Corning) coated glass coverslips (12 mm, #1.5; ref # 64–0732, Warner Instruments, Camden, CT). Neurons were plated at 150,000 cells/well in 24-well plates and were maintained in a humidified 37 °C incubator with 5% CO_2_ until they were used for imaging at DIV 21–25.

### Neuronal transfection

Neurons were transfected at DIV 3 as previously described [24,25] using Lipofectamine 2000 (cat#: 11668027, Thermo Fisher Scientific). EGFP, under the control of a human ubiquitin promoter (pFUg-EGFP), was used as a cell-filling dye to visualize neuronal morphology [24,25]. For each well of a 24-well plate, the conditioned medium was first collected from plated neurons and replaced with 300 µL of Neurobasal medium without any supplements. About 100 µL of transfection mix containing 0.5 µL of Lipofectamine 2000 and 200 ng of pFUg-EGFP plasmid was then added to each well of a 24-well plate. Neurons were incubated with the transfection cocktail at 37 °C for 2 h. After 2 h, the transfection medium was replaced with 500 µL of the warmed conditioned medium that was sterilized by passing through a 0.2 µm filter (Millipore-Sigma). Transfected neurons were then placed in a humidified 37 °C incubator until DIV 21–25, at which point they were used for immunocytochemistry and STED imaging. HEK 293T cells were transfected with 250 ng of PSD-95-EGFP or SYT1-Halo, both under control of human ubiquitin promoter, constructs using calcium phosphate precipitation as previously described [74,75]. After 24 h, cells were immunostained with the specified concentrations of PSD-95 and SYT1 antibodies and nanobodies.

### Immunocytochemistry

Cultured cortical neurons were fixed between DIV 21 and DIV 25 in 4% PFA containing 2% sucrose supplemented with 0.003% (v/v) glutaraldehyde in 1× PBS for 8 min at room temperature [24,25]. Fixed neurons were then washed three times in 1× PBS. Coverslips were next blocked and permeabilized for 1 h at room temperature in 1% ovalbumin (ref # A5503, Millipore-Sigma) and 0.2% gelatin from cold-water fish (ref # G7041, Millipore-Sigma) in 1× PBS containing 0.1% saponin (ref # 558255, Millipore-Sigma). Neurons were immunostained for 2 h at room temperature or overnight at 4 °C with the indicated primary antibodies or nanobodies, washed three times in 1× PBS, and then exposed to corresponding secondary antibodies (if needed) for 1 h at room temperature. After washing three times in 1× PBS, coverslips were mounted with ProLong Glass Antifade mounting medium and used for confocal and STED imaging after 24–48 h, when the mounting medium was fully cured.

### Confocal imaging and analysis

Cryosections collected from Thy-1-YFP-H mouse somatosensory cortex (S1) were imaged by confocal scanning microscopy using a Leica Stellaris 8 confocal and STED microscope system (Leica Microsystem, Mannheim, Germany). Confocal images were acquired with a 100× oil immersion objective (1.4 Numerical Aperture (NA), Leica) at 1.7–1.9 zoom to obtain a 100 nm pixel size. Stacks were the collection of 10−12 images taken at 0.3 µm intervals and were analyzed using ImageJ as maximum intensity projections with the experimenter blinded to treatment conditions. For each experimental condition, images were taken from at least three different Thy-1-YFP-H animals.

### Puncta co-clustering analysis

Analyses were performed blinded to the experimental condition (treatment of brain tissue). Individual pre- and post-synaptic puncta and colocalized clusters were analyzed using ImageJ custom-build macros designed to automatically count the number and size of clusters along GFP-labeled dendrites as described previously [74–77]. Each channel was thresholded to the mean +  2 × standard deviation (SD) and converted to a binary image. Individual pre- and post-synaptic puncta were defined as continuous clusters of 3–100 pixels (the size of each pixel was 100 nm) along at least 50 µm of dendrite. For the colocalization analysis, co-clusters were defined as > 1-pixel overlap between binarized pre- and post-synaptic clusters from two different channels.

### STED imaging

#### Beam alignment.

Before each experiment and at least once per hour during image acquisition, we aligned the excitation and STED lasers using auto-alignment. To ensure precise alignment in the Stellaris 8 STED system, we used 80 nm gold beads as alignment markers. Briefly, we set the excitation laser to a low power (<1%) to prevent heating of the beads, while the STED laser was also set to a low power (<0.5%) sufficient to form a visible donut. Using HyDS detectors in reflection mode, we focused on a single bead and ensured that the excitation beam passes through the center of the STED beam’s donut, maintaining an overlap of less than 25 nm between the excitation beam and the inner edge of the STED donut (S10A Fig). We further verified alignment by measuring the offset in the X and Y directions using a standard Leica STED slide with anti-nuclear pore labeling stained with the Abberior STAR 635P fluorophore, confirming alignment accuracy (S10B, S10C Fig).

#### Image acquisition.

Two-color tau-STED imaging of endogenous proteins was performed on fixed neurons in cell culture and 6 µm brain sections collected from Thy-1-YFP-H mice based on protocols developed previously [24,25]. A Leica Stellaris 8 3D tau-STED confocal and super-resolution system (Leica Microsystem) equipped with a tunable white light laser (WLL), CW 592 nm, CW 660 nm, and pulsed 775 nm STED depletion lines were used for image acquisition. HyD-X detectors set to photon counting mode at a 12 mV threshold were used to capture single photons for STED image acquisition. The 100× oil immersion objective (1.4 NA) with 4.5× digital zoom to obtain ~20 nm pixel size was used to acquire image stacks at 150 nm intervals using 400 Hz scanning of 1,024 × 1,024-pixel imaging fields (~20 µm × 20 µm). Line accumulations (2× – 3×) were used to image individual channels. Stacks for each channel were acquired in a sequential mode using the “between stacks acquisition” setting. Target proteins labeled with nanobodies or antibodies (S1 Table) were acquired using the Fast Lifetime Contrast (FALCON) enabled tau-STED module (Leica) with the time-gate on HyD-X detectors adjusted between 0.1 and 6 nanoseconds. Abberior STAR 635P/Atto 647N labeled endogenous proteins were excited with the 635 nm laser (10%–15% maximal AOBS laser power). Alexa Fluor 594/Abberior STAR 580 labeled endogenous proteins were excited using the 594 nm laser (5%–12% maximal AOBS power). The pulsed 775 nm STED depletion line set at 10% of maximal AOBS laser power was used for Abberior STAR 635P/Atto 647N fluorophores and 15% of maximal AOBS laser power for Alexa Fluor 594/Abberior STAR 580 fluorophores to generate STED. Using these settings, we were able to obtain an XY resolution of ~50 nm, measured by FWHM of GATTA STED nano-rulers (STED 50R Brightline, GATTAquant, GMBH, Munich, Germany, S6 Fig) [24]. VGluT1 labeled with AlexaFluor-555 or Atto 542 were excited using 555 nm laser (10% AOBS). The CW 660 nm line (12% AOBS) was used to generate STED. All data shown were imaged using 3D STED with 20% STED laser power re-directed toward the Z-donut, which allowed us to resolve GATTA 3D STED nano-rulers spaced 90 nm along the Z-axis (STED 3D 90R, GATTAquant, S6 Fig). For simultaneous confocal/STED imaging, fluorophores that were not exposed to STED lasers (EYFP, Alexa Fluor 790, Atto 542) were imaged first, using only excitation lines provided by the WLL. This was then followed by STED acquisition of Abberior STAR 635P fluorophore and then Alexa Fluor 594 fluorophores, all in the same sequence.

### STED image processing

Following image acquisition, background photons were removed from raw STED images by adjusting tau strength using the tau-STED module in the FALCON application on the Stellaris 8 STED instrument (Leica Microsystems). The ability to remove unwanted photons relies on generating FLIM profiles of every photon captured by HyD-X detectors [48]. The tau strength was adjusted to reduce blur surrounding cluster edges in a manner that did not distort existing clusters or create cluster artifacts. With 5–10 captured photons per pixel, this usually resulted in a tau strength between 100–200. Once the tau strength was determined for each STED channel, it was kept constant throughout all experiments. Afterward, tau-STED images were subjected to 0.8-pixel Gaussian blur, and brightness was adjusted to generate high-contrast images for figures and analysis. None of the images acquired were deconvolved. Tau-STED images were obtained as 16-bit Tiff files. These images were initially adjusted in ImageJ by subtracting the background (mean pixel intensities of the entire 1,024 × 1,024 frame) from every channel. We then converted these 16-bit files to 8-bit images that were subsequently used for 3D reconstructions in the Neurolucida 360 software (MBF Bioscience, Williston, VT). Only those post-synaptic clusters that localized with the spine head and associated (colocalized) Bassoon clusters are shown in the rendered images. The rest of the clusters were omitted for enhanced clarity [24,25].

### STED image analysis

All analyses were conducted offline using Fiji ImageJ and built-in macros, as described below. Neurolucida 360 (MBF Bioscience) was used to reconstruct confocal and STED images in 3D and obtain parameters pertaining to individual clusters and spines.

#### Nanomodule identification.

Super-resolution analysis of synaptic cluster localizations in dendritic spines was performed on a per-spine basis as described [24,25]. Individual spines were converted to binary masks by thresholding YFP (enhanced by GFP immunolabeling) to the mean + 2 × SD of the 1,024 × 1,024-pixel area corresponding to the entire image field. The localization of PSD-95 and Bassoon NCs was then determined per spine using two different approaches. First, regions of interest (ROIs) of each thresholded spine head were used to manually assign STED-resolved puncta to spines. PSD-95 clusters were assigned to a spine if the thresholded pixel areas were entirely within the spine head ROI. Bassoon clusters were assigned to a spine if the thresholded pixel areas either entirely or partially overlapped with the spine head ROI. Spine ROI colocalization with each cluster was evaluated independently for each Z-section. Mean +  1.5 SD of fluorescence intensities within the spine ROI was used to identify discrete PSD-95 and Bassoon NCs [24,25]. This criterion was applied as a standard for our STED imaging, including the calibration of STED resolution using GATTA bead nano-rulers (S6 Fig) [24]. Orthogonal views of overlaid image stacks were used to verify that individual clusters colocalized with spine ROI in the Z plane. Following the NC assignment, we categorized spines either as VGluT1 or VGluT2 based on their colocalization ( >1 pixel overlap) with YFP-labeled dendritic spines. Sometimes, both VGluT1 and VGluT2 appeared to colocalize with the same spine. In those cases, we used the colocalization of aligned nanoscale-resolved Bassoon and PSD-95 with either VGluT1 or VGluT2 to determine under which glutamatergic marker to categorize spines with apparent dual innervation.

In our second approach, we used the 3D visualization and analysis software Neurolucida 360 (MBF Bioscience) to independently verify the 3D segmentation of individual NCs by reconstructing confocal and tau-STED channels. We converted 16-bit images to 8-bit Tiff files by assigning the value of 255 to the brightest pixel in each 16-bit image. We then reconstructed dendritic trees by tracing YFP-filled dendrites using the Rayburst crawl mode [78]. Spines were detected automatically by adjusting the outer range to a 2.5 µm maximum value, a minimum height of 0.3 µm, a detector sensitivity of 50%−150%, and a minimum count of 10 voxels. These values were sufficient to reconstruct most spines. A minority of spines had to be reconstructed manually by adjusting the above values. PSD-95 and Bassoon NCs were reconstructed in 3D using detector settings in Neurolucida 360 with a diameter of 0.1–0.5 µm and a sensitivity set to 200%–300%. Clusters were reconstructed only if they contained a minimum of 10 contiguous voxels. 3D-resolved PSD-95 NCs were then assigned to individual spines in a semi-automatic manner by setting the proximity value between NCs and spine heads to 0.01 µm (inside the spine). For Bassoon, this value ranged from 0.15–0.2 µm. Some clusters had to be reconstructed manually by increasing either detector diameter, sensitivity, or both. Reconstructed images were then exported to the associated 3D Neurolucida Explorer (MBF Bioscience) to obtain the measurements (volumes and NC numbers per spine) for 3D reconstructed clusters.

#### 3D STED cluster distance analysis.

Colocalization and nearest-neighbor analysis of 3D STED-resolved synaptic clusters were performed on a per-cluster basis using the entire area of an image (1,024 × 1,024-pixel format). Segmentation and subsequent measurements of distances of segmented clusters were performed using the DiAna plug-in in ImageJ that enabled the analysis to be done in an automated way [50]. 3D-resolved NCs of synaptic proteins (acquired in tau-STED super-resolution) were identified by binarizing each channel separately using intensity thresholds followed by segmentation of individual clusters in the DiAna segmentation window [24]. Segmentation of all synaptic clusters was performed using the local maxima method combined with user-defined thresholds [24,50]. Local maxima were identified using a radius of five pixels (100 nm) in the XY plane and two pixels (300 nm) in the Z plane. Local thresholds were defined by thresholding individual 16-bit images to the mean +  2 × SD of intensity values in an entire 1,024 × 1,024 image frame. The maximum radius of segmented clusters was set to 20 pixels (individual pixel sizes in our images were set to 20–25 nm to allow a maximum resolution of ~50 nm). The SD for Gaussian fit and threshold calculation was set to 1.5. Minimum and maximum voxel sizes were set to three and 20,000, respectively. Distance analysis of segmented clusters was based on classical Euclidean distance computation [50]. For measurements of the nearest neighbors and trans*-*synaptic clusters, we implemented center-to-center distances where, for each object from one image (channel 1), the center-to-center distances with all objects from another image (channel 2) were computed in 3D, and closest neighbor distances were reported [24].

### Expansion of brain tissue using Magnify

The isotropic expansion of brain cryosections using the magnify strategy was adapted from Klimas and colleagues and is briefly detailed below [56].

#### Pre-expansion antibody staining.

Brain cryosections were prepared and immunostained as outlined above in the “Brain Tissue Collection and Processing” and “Immunohistochemistry of Non-Treated Cryosections” sections. After labeling with VGluT1, VGluT2, and GFP, the sections were stored in 1× PBS prior to the subsequent steps.

#### In situ polymer synthesis of samples with magnify.

A monomer solution made of 4% DMAA (v/v), 34% SA (w/v), 10% AA (w/v), 0.01% Bis (w/v), 1% NaCl (w/v), and 1 × PBS was prepared and stored at 4 °C before synthesis. Excess PBS around the tissue was absorbed with a Kimwipe, and sections were allowed to air dry partially on the slide. Immediately before gelation, the chemicals 4HT, TEMED, APS, and methacrolein were added to the gel monomer solution to a final concentration of 0.25% (w/v) APS, 0.001% 4HT (w/v, mouse brain and organoid only), 0.04% TEMED (v/v), and 0.1% (v/v) methacrolein, adding TEMED and APS last to prevent premature gelation. Tissue was incubated in gelling solution for 30 min at 4 °C to allow the monomer solution to diffuse into the tissue. A glass microscope slide was placed backside down over the gelling chamber, and the samples were incubated overnight in a humidified container at 37 °C to complete gelation.

#### Sample digestion and expansion with magnify.

After gelation, the glass slide cover was removed from the gelling chamber, blank gel surrounding the tissue was trimmed from the samples, and the tissue was cut into smaller pieces if necessary. Samples were then incubated in homogenization buffer (1%–10% w/v SDS, 8 M Urea, 25 mM EDTA, 2× PBS, pH 7.5 at RT) for 9 h at 90 °C with shaking. Homogenized samples were then washed three times with 1× PBS at RT, followed by at least three washes in 1% decaethylene glycol monododecyl ether (C12E10)/1× PBS or 1% PBST at RT or 60 °C to remove remaining SDS. Expanded brain samples were additionally incubated in 1% decaethylene glycol monododecyl ether (C12E10)/1× PBS at 60 °C for 1 h. Samples were finally washed an additional three times for at least 10 min each with 1× PBS at RT and stored in 1× PBS containing 0.02% sodium azide at 4 °C.

#### Post-expansion immunostaining of mouse brain samples.

Expanded samples were taken from storage (1× PBS +  0.02% sodium azide) at 4 °C and washed three times for 10 min each in 1× PBS at RT. Samples were then incubated with primary antibodies in 1% TritonX-100 in 1× PBS overnight at RT. Samples were washed three times for at least 20 min each in 1× PBS at RT before incubation in 1% TritonX-100 in 1× PBS with the corresponding secondary antibodies for 3 h at RT. Before imaging, samples were washed with 1× PBS. After staining, samples were washed in water for at least 10 min. This was repeated until the sample was fully expanded—at least three exchanges of water.

### Imaging of expanded brain tissue with Crest Deep SIM system (Ex-SIM)

Images were acquired on a Nikon Ti inverted microscope equipped with an N40XLWD-NIR – 40X Nikon CFI APO LWD NIR Objective, 1.15 NA, 0.59–0.61 mm WD and CrestOptics X-Light V3 Spinning Disk and Deep SIM scan head with a Photometrics Kenetix sCMOS camera. Optical sections spaced at 0.3 µm were used to acquire 40 µm stacks of YFP fluorescent dendrites along with VGluT1 and VGluT2 puncta. Images were analyzed using the same criteria as described above for nanomodule identification of STED-resolved clusters.

### Experimental design and statistical analyses

Data were acquired and analyzed based on the standards in the field; however, no method of biological specimen randomization was used to determine how samples were allocated to experimental groups and processed. Unless otherwise stated, data are expressed as means ±  SEM. All data points collected were included for analysis. Statistical significance of the differences among groups was determined by a one-way analysis of variance followed by post hoc tests described in individual figure legends. When testing differences between two conditions, we performed a two-tailed Student’s *t* test. Kolmogorov–Smirnov (K–S) tests were used to test differences between non-parametric probability distributions. *P* values less than 0.05 were considered statistically significant. For *p* values less than 0.0001, we provide a range and not the exact number (e.g., *p* < 0.0001). The data distribution was assumed to be normal, but this was not formally tested. Sample sizes were determined based on previous publications [24,25,74–77]. All statistical analyses were performed using Graph Pad Prism statistical software (https://www.graphpad.com). Unless stated otherwise, statistical tests were conducted on a per-spine basis. Statistical tests for center-to-center distances between 3D STED clusters were performed on a per-cluster basis. Data were collected from a minimum of three independent transfection experiments or at least two different adult mice between ages P35–P90. The source data used to generate the figures and conduct statistical analyses can be found in S2 Table. Details about each statistical test performed can be found in S3 Table.

### Biological reagents

All nanobodies, primary and secondary antibodies used to immunostain brain sections and cultured neurons are listed in S1 Table. Dilution and specificity of all antibodies used were profiled in prior publications and were reported to be specific [24,25,74–77]. PSD-95 nanobody has been shown not to cross-react with mouse and rat PSD-93, SAP-97, and SAP-102 [46]. VGluT1 specificity was tested in the current study by determining colocalization with antibodies for VGluT1 and VGluT2 (S3 Fig). SYT1 specificity and affinity were determined in [47].

## Supporting information

S1 Fig Variable labeling of putative synapses by antibodies in PFA-fixed brain cryosections. **(A)** Representative confocal images of L5 apical dendrites in 6 µm cryosections collected from Thy1-YFP-H mice. Cryosections were either directly used for immunolabeling with antibodies against PSD-95 (magenta), VGluT1 (yellow), and Bassoon (cyan) to visualize pre- and post-synaptic specializations on YFP-labeled dendrites (gray, enhanced by GFP antibody) or subjected to antigen retrieval and/or CUBIC clearing. Arrows indicate PSD-95 immunolabeling in dendritic spines. Scale bar, 5 µm. **(B-D)** Quantification of PSD-95 (**B**), VGluT1 (**C**) and Bassoon (**D**) cluster densities along YFP-labeled apical dendrites in no treatment (NT, *n* =  27 neurons), Antigen Retrieved (AR, *n* =  27 neurons), CUBIC (C, *n* =  27 neurons) and CUBIC + Antigen Retrieved (C + AR, *n* =  30 neurons) conditions (*p < 0.01, **p < 0.007, and ***p < 0.0001, one-way ANOVA, Tukey’s post hoc). Dotted horizontal lines show comparisons to average spine densities (**p =  0.005, ***p < 0.0001, one-way ANOVA, Dunnett’s post hoc). **(E)** Quantification of dendritic spine densities (*p* =  0.5556, one-way ANOVA, Tukey’s post hoc). **(F, G)** Quantification of colocalized PSD-95 and VGluT1 (*p* =  0.7983, one-way ANOVA) or PSD-95 and Bassoon (*p < 0.05, one-way ANOVA, Tukey’s post hoc) cluster densities. Average spine density (dotted lines from **E**) comparisons to colocalized clusters in **F, G** (***p < 0.0001, one-way ANOVA, Dunnett’s post hoc). Bar graphs represent the mean ±  SEM. For each condition, data were collected from a minimum of 10 different neurons (dots on graph) across brain sections acquired from at least three biological replicates. The source data for panels B-G can be found in S2 Table.
(TIF)

S2 Fig Colocalization of pre- and post-synaptic clusters detected with antibodies and nanobodies in PFA-fixed cryosections. **(A)** Percent colocalization between PSD-95 and VGluT1 labeled by antibodies under various tissue post-treatment conditions (detailed in S1 Fig) compared to nanobody labeling in non-treated (NT, Fig 1) cryosections (*p =  0.0075, **p =  0.001, ***p <  0.0001, one-way ANOVA, Dunnett’s post hoc). **(B)** Percent colocalization between PSD-95 and Bassoon labeled by antibodies under various tissue post-treatment conditions (detailed in S1 Fig) compared to nanobody labeling in non-treated (NT, Fig 1) cryosections (**p =  0.001, ***p <  0.0001, one-way ANOVA, Dunnett’s post hoc). Bar graphs represent the mean ±  SEM. For each condition, data were collected from a minimum of 10 different neurons (dots on graph) across brain sections acquired from at least three different male and female Thy1-YFP-H mice. The source data for panels A and B can be found in S2 Table.
(TIF)

S3 Fig VGluT1 nanobody is specific for VGluT1 but not for VGluT2. **(A)** Representative three-channel confocal images of nanobody-labeled VGluT1 (Atto 542, green), antibody-labeled VGluT1 (Alexa Fluor 594, red), and antibody-labeled VGluT2 (Atto 647N, red) clusters in S1. **(B)** Percent colocalization between nanobody-labeled VGluT1 clusters and antibody-labeled VGluT1 (a-VG1) or VGluT2 (a-VG2) puncta (*n* =  10 images, ***p < 0.0001, unpaired Student’s *t* test). Bar graphs represent mean ±  SEM. Dots on bar graphs represent data from individual images collected from at least three independently immunostained brain sections. Scale bar, 5 µm. The source data for the panel B can be found in S2 Table.
(TIF)

S4 Fig Glyoxal fixation enhances the detection of PSD-95 labeling with antibodies. **(A, B)** Confocal images of L5 apical dendrites in 6 µm cortical sections from Thy-1-YFP-H mice. Antibodies (right panels) and nanobodies (left panels) were used to detect PSD-95 (magenta), Synaptotagmin-1 (SYT1, yellow), or VGluT1 (VG1, yellow). Bassoon (cyan) was labeled with the antibody. Arrows indicate dendritic spines. Scale bar for **A** and **B**: 5 µm. **(C-F)** Quantification of puncta densities along YFP-labeled dendrites using: (**C**) PSD-95 nanobody (*n* =  42 neurons) or two antibodies (IgG1: *n* =  21, IgG2A: *n* =  18, *p < 0.05, one-way ANOVA, Tukey’s post hoc), (**D**) VGluT1 nanobody (*n* =  23 neurons) or antibody (*n* =  18 neurons, ***p < 0.0001, unpaired Student’s *t* test), (**E**) SYT1 nanobody (*n* =  21 neurons) or antibody (*n* =  19 neurons, ***p < 0.0001, unpaired Student’s *t* test), (**F**) Bassoon antibody (*n* =  41 neurons). **(G)** Colocalized cluster densities and percentage of colocalization of PSD-95 with Bassoon along YFP-labeled dendrites using PSD-95 labeling with either a nanobody or two antibodies (*p =  0.0393, **p =  0.0016, ***p < 0.0001, one-way ANOVA, Tukey’s post hoc). **(H)** Colocalized cluster densities and percentage of colocalization of PSD-95 with VGluT1 along YFP-labeled dendrites (**p =  0.0012, ***p < 0.0001, unpaired Student’s *t* test). **(I)** Colocalized cluster densities and percentage of colocalization of PSD-95 with SYT1 along YFP-labeled dendrites (***p < 0.0001, *p* =  0.087, unpaired Student’s *t* test). Bar graphs represent means ±  SEM obtained from the indicated number of neurons (dots) from two different glyoxal-perfused mice. The source data for panels C-I can be found in S2 Table.
(TIF)

S5 Fig Nanobodies bind to expressed proteins over a broad range of concentrations. **(A)** Maximum projection images of HEK 293T cells co-transfected with RFP and PSD-95-GFP and immunostained with the indicated concentrations of PSD-95 nanobody or two different PSD-95 antibodies. **(B)** Quantification of average PSD-95 fluorescence intensity of PSD-95-GFP^ +^ HEK cells across indicated concentrations (*p < 0.0001, one-way ANOVA, Tukey’s post hoc, nanobody: *n* =  274 cells [20 nM], 375 [10 nM], 402 [5 nM], 406 [2.5 nM], 251 [0.25 nM]; IgG2A: *n* =  163 cells [20 nM], 175 [10 nM], 166 [5 nM], 140 [2.5 nM], 137 [0.25 nM]; IgG1: 300 cells [20 nM], 372 [10 nM], 238 [5 nM], 233 [2.5 nM], and 243 [0.25 nM] from 2–4 independent transfection experiments). **(C)** Maximum projection images of HEK 293T cells co-transfected with RFP and SYT1. The cells were immunostained with the indicated concentrations of SYT1 nanobody or antibody. Scale bar for all images in **A** and **C**: 10 µm. **(D)** Quantification of average SYT1 fluorescence intensity in all RFP transfected cells per image across the reagent concentrations (***p =  0.0009, **p =  0.0002, *p =  0.0298, unpaired Student’s *t* test; nanobody: *n* =  7 images [20 nM], 9 [10 nM], 12 [5 nM], 11 [2.5 nM], 9 [0.25 nM]; antibody: *n* =  9 images [20 nM], 6 [10 nM], 10 [5 nM], 11 [2.5 nM], and 10 [0.25 nM] from three independent transfection experiments). Graphs represent mean ±  SEM. The source data for panels B and D can be found in S2 Table.
(TIF)

S6 Fig Calibration of 3D tau-STED resolution. **(A)** A representative image of GATTA 50 ±  5 nm laterally spaced Atto 647N-labeled nano ruler beads imaged in confocal (red) and 3D tau-STED (green). Square indicates a single GATTA bead, shown in a larger view in confocal and STED channels on the right. Scale bars, 1 µm (left), 100 nm (inset, right). **(B)** Confocal and tau-STED line profiles through individual GATTA beads. Red line shows the mean (54 nm, *n* =  43 beads, gray lines) with Rayleigh criterion of 13%. **(C)** Average peak-to-peak distances calculated from line profiles of 50 ±  5 nm spaced GATTA beads imaged in 2D (0% Z-depletion, *n* =  29 beads) and 3D (20% Z-depletion, *n* =  43 beads, *p* =  0.0697, unpaired Student’s *t* test). **(D, E)** Representative confocal and 3D tau-STED images of dual-labeled GATTA bead nano-rulers and associated line profiles (*n* =  60 beads, gray lines). Beads labeled with Atto 647N are spaced at 140 ±  5 nm, while Atto 647N and Atto 594 beads are spaced at 70 ±  5 nm. Green and red lines represent means (Rayleigh criterion of 33%). Scale bar: 250 nm. **(F)** Representative image of 3D GATTA 90 ±  5 nm axially spaced Atto 647N-labeled nano ruler beads imaged in 3D tau-STED. The bottom image shows orthogonal *xz* views of beads # 1–4. Scale bar, 1 µm. **(G)** Line profiles of beads # 1–4 in the *xz* view (shown in **F**) demonstrating peak-to-peak separation of 90 to 160 nm. **(H)** Zoomed-in view of the bead # 4 and associated orthogonal (*xy* and *xz*) projections. Scale bar, 500 nm. **(I, J)** 3D Neurolucida 360 reconstruction of GATTA beads from **F**. Beads #3 and #4 are shown. Inset (**J**) shows zoomed-in view of bead # 4. **(K)** Average nearest-neighbor distances calculated from Neurolucida 360 reconstructions of 90 ±  5 nm axially spaced GATTA beads (*d* =  93.6 ±  2.9 nm, *n* =  133 beads). Mean (red line) with individual GATTA beads (dots) are shown. **(L)** Average volume of single GATTA beads calculated from 3D Neurolucida 360 reconstructions of 90 ±  5 nm axially spaced GATTA beads (*v* =  0.0012 ±  0.0001 µm^3^, *n* =  136 beads). Mean (red line) with individual GATTA bead volumes (dots) are shown. The source data for panels C, K, and L can be found in S2 Table.
(TIF)

S7 Fig STED imaging reveals differences in the FWHM of synaptic clusters labeled with nanobodies or antibodies. **(A)** 3D tau-STED images of PSD-95 clusters in DIV 21 cortical cultures stained with either a mouse primary and Atto 647N-conjugated secondary antibody (red) or a PSD-95-specific nanobody (Abberior STAR 635P, green). Images were acquired separately from sister cultures with the same settings. Scale bar for **A, D, G**: 3 µm. **(B)** Cumulative frequency distributions of FWHM for tau-STED-resolved PSD-95 nanoclusters labeled with antibody (red, *n* =  214) and nanobody (green, *n* =  275; ***p <  0.0001, Kolmogorov–Smirnov test). **(C)** Average FWHM per image for PSD-95 nanoclusters labeled with antibody and nanobody (*n* =  4 images, ***p < 0.0001, unpaired Student’s *t* test). **(D)** 3D tau-STED images of SYT1 clusters in DIV 21 cortical cultures stained with either a mouse primary and Atto 647N-conjugated secondary antibody (red) or a SYT1-specific nanobody (Abberior STAR 635P, green). Images were acquired separately from sister cultures using the same STED settings. **(E)** Cumulative frequency distributions of FWHM for tau-STED-resolved SYT1 nanoclusters labeled with antibody (red, *n* =  322) and nanobody (green, *n* =  333; ***p <  0.0001, Kolmogorov–Smirnov test). **(F)** Average FWHM per image for SYT1 nanoclusters labeled with antibody and nanobody (*n* =  4 images, **p =  0.0006, unpaired Student’s *t* test). **(G)** 3D tau-STED images of VGluT1 nanoclusters in DIV 21 cortical cultures labeled with either a mouse primary and Alexa 555-conjugated secondary antibody (red) or a VGluT1-specific nanobody (Atto 542, green). Images were acquired separately from sister cultures using the same STED settings. **(H)** Cumulative frequency distributions of FWHM for tau-STED-resolved VGluT1 nanoclusters labeled with antibody (red, *n* =  160) and nanobody (green, *n* =  86; ***p <  0.0001, Kolmogorov–Smirnov test). **(I)** Average FWHM per image for VGluT1 nanoclusters labeled with antibody and nanobody (*n* =  3 images, **p =  0.0006, unpaired Student’s *t* test). Bar graphs represent means ±  SEM obtained from at least three different images (dots) from two independent cortical cultures. The source data for panels C, F, and I can be found in S2 Table.
(TIF)

S8 Fig Trans-synaptic alignment of PSD-95 and Bassoon nanomodules in the XY and Z planes. **(A)** A representative two-color STED image of PSD-95 (magenta) and Bassoon (cyan) nanoclusters in layer 5 (L5). White arrows indicate puncta aligned in the XY plane (Type A), while yellow arrows point to puncta aligned in the Z plane (Type B). Orthogonal views of Type A nanoclusters (white square) are shown on the right, and orthogonal views of Type B nanoclusters (yellow square) are displayed below. Scale bars: 2 µm, orthogonal views 200 nm. **(B)** Quantification of center-to-center distances between PSD-95 and Bassoon for Type A and Type B trans*-*synaptic nanoclusters (*p* =  0.8258, unpaired Student’s *t* test). Each dot represents an individual Type A (*n* =  72) or Type B (*n* =  59) trans*-*synaptic PSD-95/Bassoon nanocluster pair. **(C)** Densities of YFP-labeled spines on dendrites of L5 neurons, projecting in XY and Z orientations. Dots represent average spine densities for the indicated subtypes in apical (L2/3) and basal (L5) dendrites (*n* =  20 dendritic segments, ***p <  0.0001, unpaired Student’s *t* test). **(D)** Density of XY (Type A) and Z (Type B) projecting YFP-labeled spines along apical dendrites (Layers 2/3) from Thy-1-YFP-H mice. Dots represent average spine densities across 10 different dendritic segments (***p <  0.0001, unpaired Student’s *t* test). **(E)** Density of Type A and Type B projecting YFP-labeled spines along basal dendrites (Layer 5) from Thy-1-YFP-H mice. Dots represent average spine densities across 10 different dendritic segments (***p <  0.0001, unpaired Student’s *t* test). Bar graphs represent means ±  SEM collected from two Thy-1-YFP-H mice. The spread of the data, indicated by dots on the bar graphs, is defined above. The source data for panels B-E can be found in S2 Table.
(TIF)

S9 Fig Confirmation of VGluT cluster assignment by the expansion of proteome with Magnify. **(A)** A representative image of L5 pyramidal neuron apical dendrites in an expanded 5 µm cryosection from Thy-1-YFP H mouse S1 cortex imaged using deep SIM microscopy (Ex-SIM). Antibodies were used to recover and detect VGluT1 (magenta) and VGluT2 (cyan) after proteome expansion to assess their colocalization with dendritic spines (YFP, gray enhanced by GFP immunolabeling) in X, Y, and Z planes. Orthogonal views indicate the non-overlapping localization of VGluT1 and VGluT2 clusters in the Z plane. Scale bar (XY): 20 µm. **(B)** High-resolution images of three spines from the inset (square) in **A.** Expansion of samples in X, Y, and Z planes allowed unambiguous VGluT1 and VGluT2 assignment to individual dendritic spines. Scale bar: 5 µm (inset ROI), 2 µm (spines). **(C)** 3D rendering of spines in **B** confirms non-overlapping VGluT1 and VGluT2 localization to individual spines. Scale bars: 2 µm. **(D)** Discrete VGluT1 (yellow arrowheads in **A**) and VGluT2 (yellow arrows in **A**) clusters on individual spines using Ex-SIM. White arrows indicate discrete nanoclusters on YFP-labeled spines. Scale bar: 5 µm. **(E)** Fraction of spines receiving VGluT1 (*n* =  264 spines) or VGluT2 (*n* =  70 spines) input. Few spines (*n* =  7) had both VGluT1 and VGluT2 clusters and three spines did not colocalize with either VGluT1 or VGluT2. **(F)** Fraction of spines with one versus multiple (two and three or more) VGluT1 or VGluT2 clusters. The source data for panels E and F can be found in S2 Table.
(TIF)

S10 Fig Verification of STED laser alignment stability. **(A)** Aligned confocal (red) and STED (green) beams visualized using light reflection from 80 nm gold particles. **(B)** Offset verification between confocal (red) and STED (green) signals using a standard Leica slide labeled with a nuclear pore antibody (Abberior STAR 635P) and imaged with a 100× 1.4 N/A STED objective. The square indicates a magnified view of the indicated clusters. The dotted lines show the direction of line profiles for offset measurement. Scale bars: 1 µm (left), 500 nm (right). **(C)** Average offsets between the centers of confocal and STED signals in the X (+6 nm, *n* =  40 clusters) and Y (−3 nm, *n* =  40 clusters) directions are below the STED resolution limit (~50 nm, see S6 Fig). The source data for the panel C can be found in S2 Table.
(TIF)

S1 Table Nanobodies and antibodies. (DOCX)

S2 Table The source data used to generate the figures and conduct statistical analyses. (XLSX)

S3 Table Details about each statistical test performed. (XLSX)

S1 Video 3D reconstruction of confocal-resolved VGluT1 and VGluT2 clusters and corresponding YFP-labeled apical dendrites. Representative 23 × 23 × 2.8 µm reconstructions of confocal channels in apical segments of L5 pyramidal neurons generated by 3D-rendering of 17 optical sections spaced at 170 nm from five-channel confocal/3D-tau-STED imaging of apical dendrites and dendritic spines on L5 pyramidal neurons in S1 of Thy-1-YFP-H mice. YFP-labeled dendrites and spines (white, enhanced by the labeling with GFP antibody), VGluT1 (red, nanobody/Atto 542), and VGluT2 (green, antibody/Alexa Flour 750) were imaged in confocal resolution. Dendrites and clusters were reconstructed using Neurolucida-360 to assign CC and TC synapses to YFP-labeled spines based on their colocalization with VGluT1 or VGluT2, respectively. Still, images of this apical dendrite and spines are shown in Fig 5.
(MP4)

S2 Video 3D reconstruction of STED-resolved PSD-95 and Bassoon in YFP-labeled apical dendrites of L5 pyramidal neurons. Representative 23 × 23 × 2.8 µm reconstructions of STED channels generated by 3D-rendering of 17 optical sections spaced at 170 nm from five-channel confocal/3D tau-STED imaging of apical dendrites on L5 pyramidal neurons in S1 of Thy-1-YFP-H mice. Dendrites and dendritic spines labeled with YFP (white, enhanced by the labeling with GFP antibody) were imaged in confocal resolution. PSD-95 (magenta, visualized by nanobody/Abberior Star 635P) and Bassoon (cyan, antibody/Alexa Fluor 594) were imaged in STED resolution. Dendrites and STED-resolved PSD-95 and Bassoon nanoclusters were reconstructed using Neurolucida-360 to determine the nano-organization of aligned pre- and post-synaptic components of active zones and PSDs in VGluT1^ +^ or VGluT2^ +^ dendritic spines. Still, images of this apical dendrite and dendritic spines with associated nanoclusters are shown in Fig 5.
(MP4)

S3 Video 3D-rendering of aligned PSD-95 and Bassoon nanoclusters (NCs) in molecularly defined YFP-labeled dendritic spines. Following the 3D reconstruction of confocal and STED channels, only clusters that colocalized with YFP-labeled dendritic spines were analyzed. Dendritic spines shown in peach color represent synapses receiving VGluT2 input. White spines represent synapses receiving VGluT1 input. Nano-organization of PSD-95 (magenta) and Bassoon (cyan) was determined by rendering tau-STED-resolved NCs in VGluT1^ +^ and VGluT2^ +^ spines. PSD-95 and Bassoon nano-architecture of all peach-colored VGluT2^ +^ spines is shown. Volumes for quantification were obtained from 3D-rendered spines and associated PSD-95 NCs. Still, images of these dendritic spines are shown in Fig 5.
(MP4)

S4 Video Deep SIM imaging of VGluT1 and VGluT2 nanoclusters and dendritic spines in expanded brain cryosections. 3D rendering of a 166 ×  166 ×  40 µm brain section from Thy1-YFP-H mice, initially sectioned at 5 µm and expanded isotropically by a factor of eight using the Magnify approach. YFP (gray) labels spine morphology, VGluT1 (magenta) identifies CC synapses, and VGluT2 (cyan) marks TC synapses. Imaging was performed using deep SIM microscopy, and the surfaces of VGluT1 and VGluT2 are shown for the spines displayed in S9D Fig.
(MOV)

## Abbreviations

ANCOVA: analysis of covariance
ANOVA: analysis of variance
CC: corticocortical
DIV: days in vitro
FALCON: Fast Lifetime Contrast
FLIM: fluorescent lifetime imaging
IHC: immunohistochemistry
NC: nanocluster
PFA: paraformaldehyde
POm: posterior-medial
PSDs: post-synaptic densities
ROIs: regions of interest
SIM: structured illumination microscopy
SYT1: Synaptotagmin-1
TC: thalamocortical
VGluT1: Vesicular Glutamate Transporter 1
WLL: white light laser
