# Supplementary figures and images for: Combining nanobody labeling with STED microscopy reveals input-specific and layer-specific organization of neocortical synapses
Source: PLoS Biol. 2025 Apr 4;23(4):e3002649. doi: 10.1371/journal.pbio.3002649 (PMC12002638; doi:10.1371/journal.pbio.3002649)

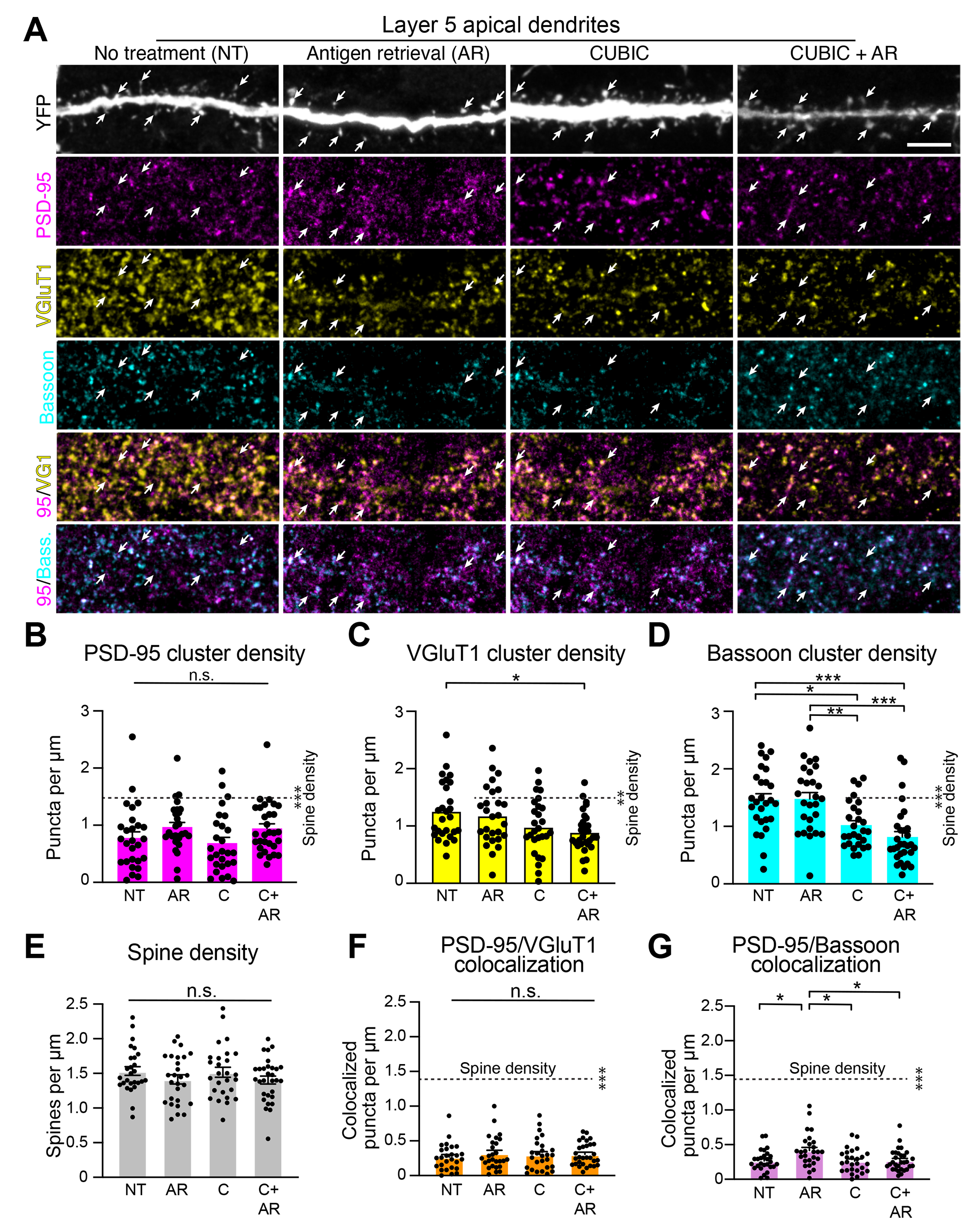

Supplement: S1 Fig — (A) Representative confocal images of L5 apical dendrites in 6 µm cryosections collected from Thy1-YFP-H mice. Cryosections were either directly used for immunolabeling with antibodies against PSD-95 (magenta), VGluT1 (yellow), and Bassoon (cyan) to visualize pre- and post-synaptic specializations on YFP-labeled dendrites (gray, enhanced by GFP antibody) or subjected to antigen retrieval and/or CUBIC clearing. Arrows indicate PSD-95 immunolabeling in dendritic spines. Scale bar, 5 µm. (B-D) Quantification of PSD-95 (B), VGluT1 (C) and Bassoon (D) cluster densities along YFP-labeled apical dendrites in no treatment (NT, n = 27 neurons), Antigen Retrieved (AR, n = 27 neurons), CUBIC (C, n = 27 neurons) and CUBIC + Antigen Retrieved (C + AR, n = 30 neurons) conditions (*p < 0.01, **p < 0.007, and ***p < 0.0001, one-way ANOVA, Tukey’s post hoc). Dotted horizontal lines show comparisons to average spine densities (**p = 0.005, ***p < 0.0001, one-way ANOVA, Dunnett’s post hoc). (E) Quantification of dendritic spine densities (p = 0.5556, one-way ANOVA, Tukey’s post hoc). (F, G) Quantification of colocalized PSD-95 and VGluT1 (p = 0.7983, one-way ANOVA) or PSD-95 and Bassoon (*p < 0.05, one-way ANOVA, Tukey’s post hoc) cluster densities. Average spine density (dotted lines from E) comparisons to colocalized clusters in F, G (***p < 0.0001, one-way ANOVA, Dunnett’s post hoc). Bar graphs represent the mean ± SEM. For each condition, data were collected from a minimum of 10 different neurons (dots on graph) across brain sections acquired from at least three biological replicates. The source data for panels B-G can be found in S2 Table. (TIF) [file pbio.3002649.s001.tif]

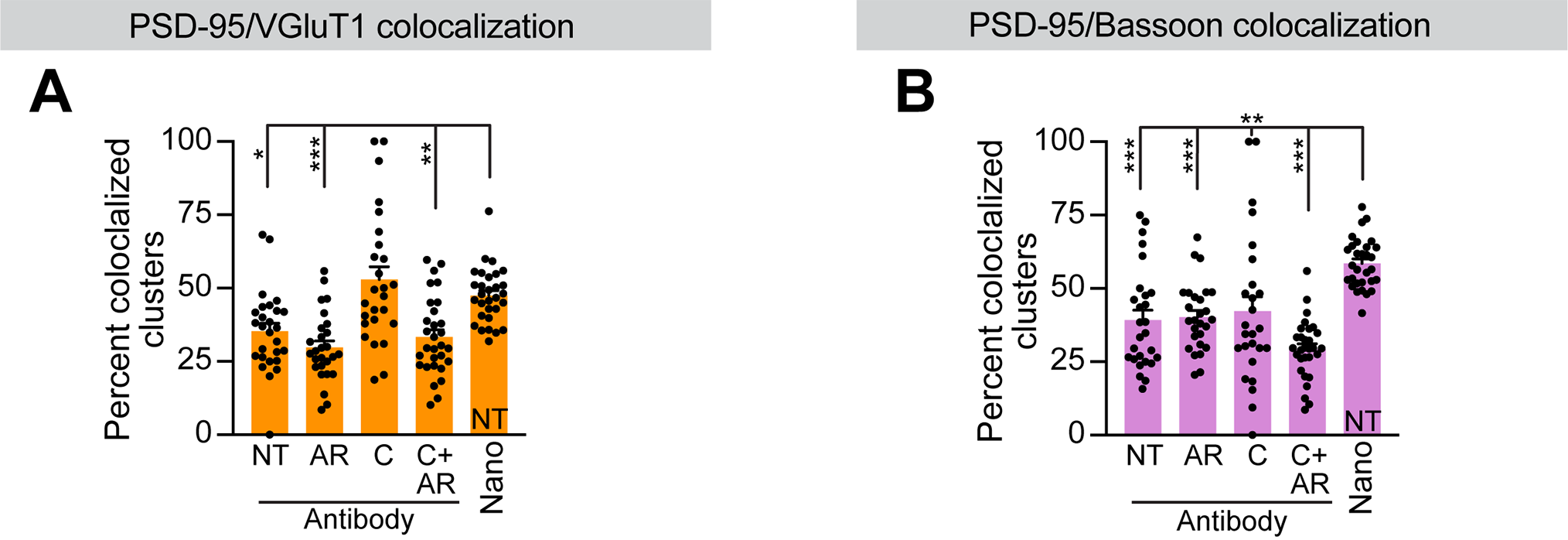

Supplement: S2 Fig — (A) Percent colocalization between PSD-95 and VGluT1 labeled by antibodies under various tissue post-treatment conditions (detailed in S1 Fig) compared to nanobody labeling in non-treated (NT, Fig 1) cryosections (*p = 0.0075, **p = 0.001, ***p < 0.0001, one-way ANOVA, Dunnett’s post hoc). (B) Percent colocalization between PSD-95 and Bassoon labeled by antibodies under various tissue post-treatment conditions (detailed in S1 Fig) compared to nanobody labeling in non-treated (NT, Fig 1) cryosections (**p = 0.001, ***p < 0.0001, one-way ANOVA, Dunnett’s post hoc). Bar graphs represent the mean ± SEM. For each condition, data were collected from a minimum of 10 different neurons (dots on graph) across brain sections acquired from at least three different male and female Thy1-YFP-H mice. The source data for panels A and B can be found in S2 Table. (TIF) [file pbio.3002649.s002.tif]

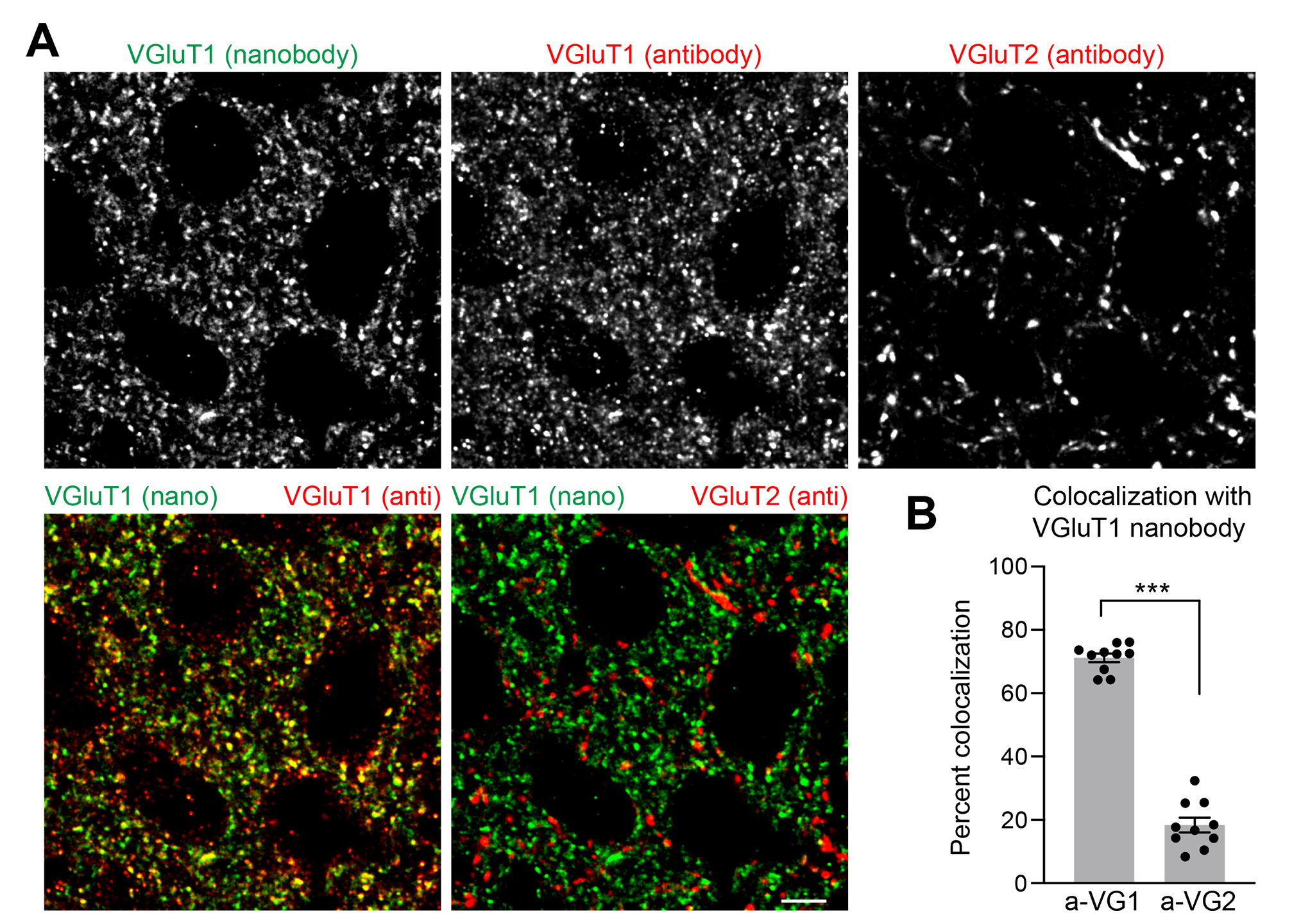

Supplement: S3 Fig — (A) Representative three-channel confocal images of nanobody-labeled VGluT1 (Atto 542, green), antibody-labeled VGluT1 (Alexa Fluor 594, red), and antibody-labeled VGluT2 (Atto 647N, red) clusters in S1. (B) Percent colocalization between nanobody-labeled VGluT1 clusters and antibody-labeled VGluT1 (a-VG1) or VGluT2 (a-VG2) puncta (n = 10 images, ***p < 0.0001, unpaired Student’s t test). Bar graphs represent mean ± SEM. Dots on bar graphs represent data from individual images collected from at least three independently immunostained brain sections. Scale bar, 5 µm. The source data for the panel B can be found in S2 Table. (TIF) [file pbio.3002649.s003.tif]

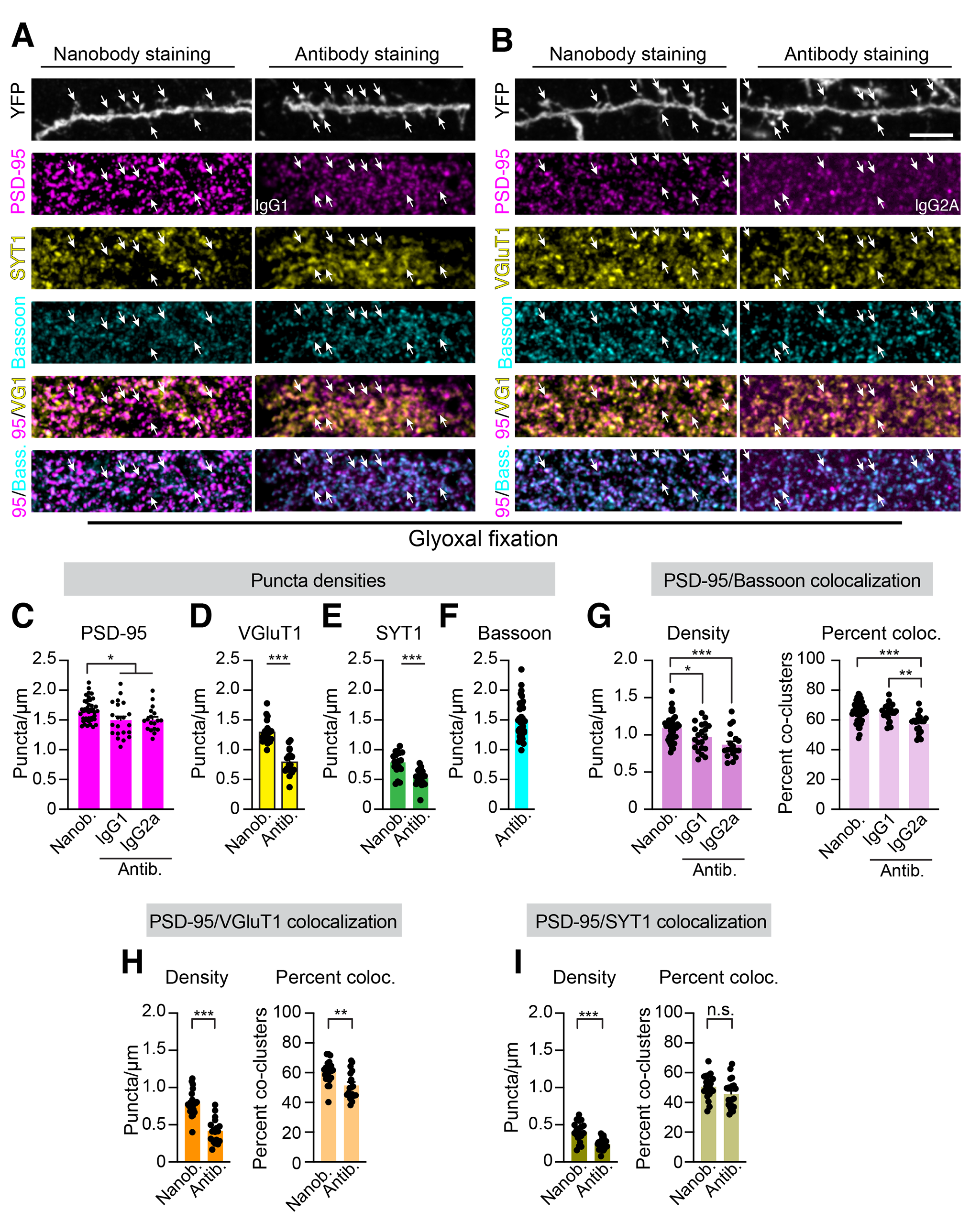

Supplement: S4 Fig — (A, B) Confocal images of L5 apical dendrites in 6 µm cortical sections from Thy-1-YFP-H mice. Antibodies (right panels) and nanobodies (left panels) were used to detect PSD-95 (magenta), Synaptotagmin-1 (SYT1, yellow), or VGluT1 (VG1, yellow). Bassoon (cyan) was labeled with the antibody. Arrows indicate dendritic spines. Scale bar for A and B: 5 µm. (C-F) Quantification of puncta densities along YFP-labeled dendrites using: (C) PSD-95 nanobody (n = 42 neurons) or two antibodies (IgG1: n = 21, IgG2A: n = 18, *p < 0.05, one-way ANOVA, Tukey’s post hoc), (D) VGluT1 nanobody (n = 23 neurons) or antibody (n = 18 neurons, ***p < 0.0001, unpaired Student’s t test), (E) SYT1 nanobody (n = 21 neurons) or antibody (n = 19 neurons, ***p < 0.0001, unpaired Student’s t test), (F) Bassoon antibody (n = 41 neurons). (G) Colocalized cluster densities and percentage of colocalization of PSD-95 with Bassoon along YFP-labeled dendrites using PSD-95 labeling with either a nanobody or two antibodies (*p = 0.0393, **p = 0.0016, ***p < 0.0001, one-way ANOVA, Tukey’s post hoc). (H) Colocalized cluster densities and percentage of colocalization of PSD-95 with VGluT1 along YFP-labeled dendrites (**p = 0.0012, ***p < 0.0001, unpaired Student’s t test). (I) Colocalized cluster densities and percentage of colocalization of PSD-95 with SYT1 along YFP-labeled dendrites (***p < 0.0001, p = 0.087, unpaired Student’s t test). Bar graphs represent means ± SEM obtained from the indicated number of neurons (dots) from two different glyoxal-perfused mice. The source data for panels C-I can be found in S2 Table. (TIF) [file pbio.3002649.s004.tif]

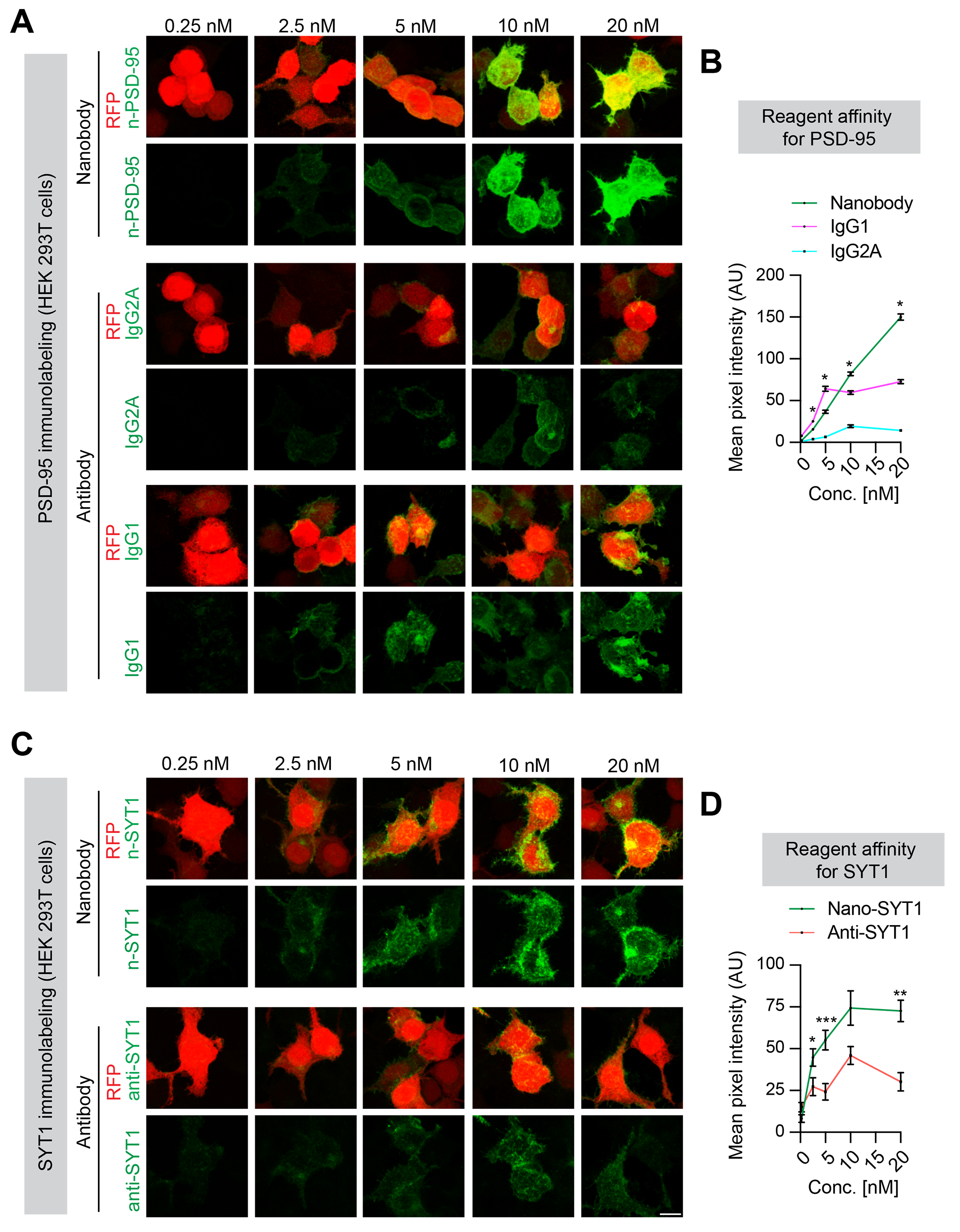

Supplement: S5 Fig — (A) Maximum projection images of HEK 293T cells co-transfected with RFP and PSD-95-GFP and immunostained with the indicated concentrations of PSD-95 nanobody or two different PSD-95 antibodies. (B) Quantification of average PSD-95 fluorescence intensity of PSD-95-GFP + HEK cells across indicated concentrations (*p < 0.0001, one-way ANOVA, Tukey’s post hoc, nanobody: n = 274 cells [20 nM], 375 [10 nM], 402 [5 nM], 406 [2.5 nM], 251 [0.25 nM]; IgG2A: n = 163 cells [20 nM], 175 [10 nM], 166 [5 nM], 140 [2.5 nM], 137 [0.25 nM]; IgG1: 300 cells [20 nM], 372 [10 nM], 238 [5 nM], 233 [2.5 nM], and 243 [0.25 nM] from 2–4 independent transfection experiments). (C) Maximum projection images of HEK 293T cells co-transfected with RFP and SYT1. The cells were immunostained with the indicated concentrations of SYT1 nanobody or antibody. Scale bar for all images in A and C: 10 µm. (D) Quantification of average SYT1 fluorescence intensity in all RFP transfected cells per image across the reagent concentrations (***p = 0.0009, **p = 0.0002, *p = 0.0298, unpaired Student’s t test; nanobody: n = 7 images [20 nM], 9 [10 nM], 12 [5 nM], 11 [2.5 nM], 9 [0.25 nM]; antibody: n = 9 images [20 nM], 6 [10 nM], 10 [5 nM], 11 [2.5 nM], and 10 [0.25 nM] from three independent transfection experiments). Graphs represent mean ± SEM. The source data for panels B and D can be found in S2 Table. (TIF) [file pbio.3002649.s005.tif]

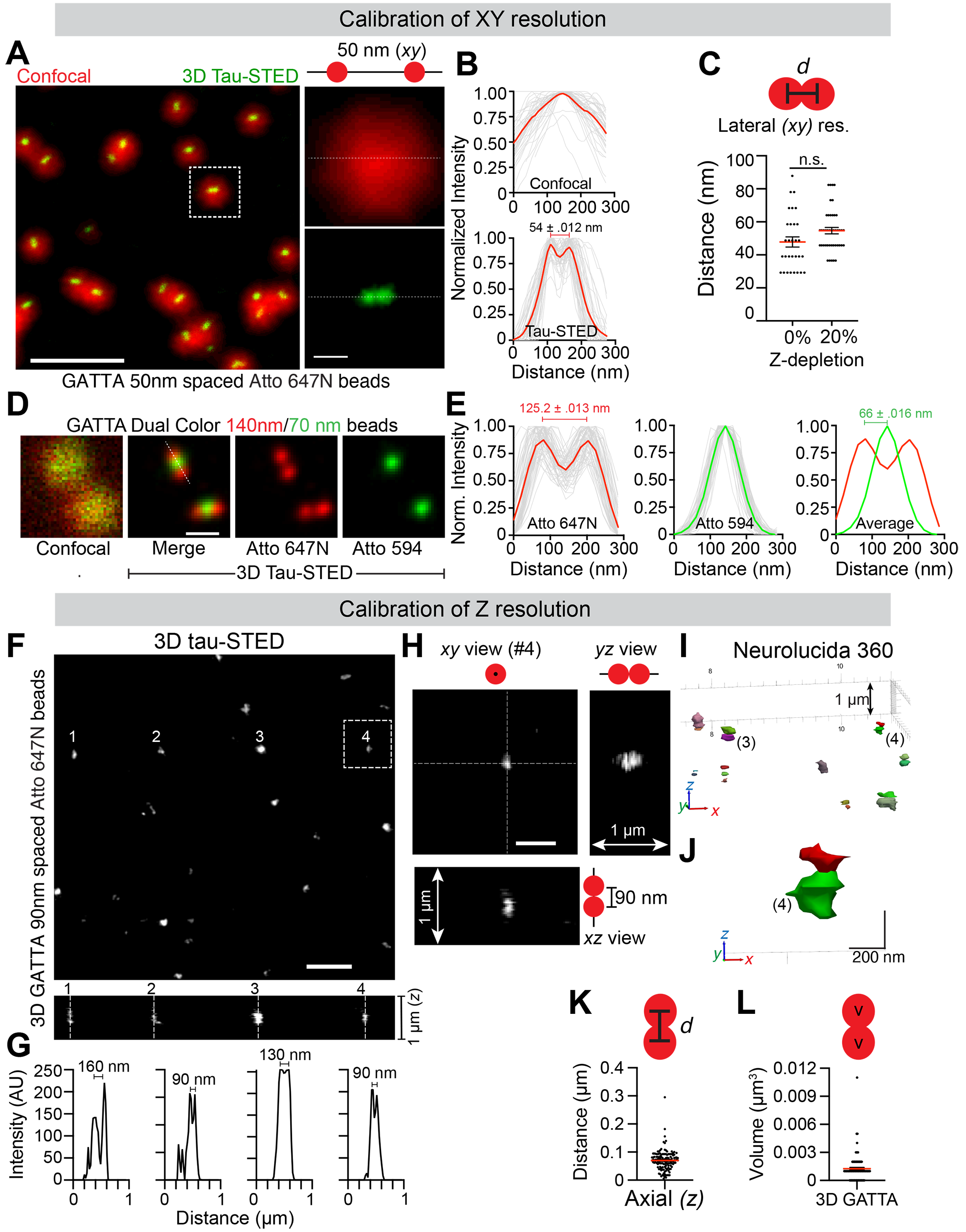

Supplement: S6 Fig — (A) A representative image of GATTA 50 ± 5 nm laterally spaced Atto 647N-labeled nano ruler beads imaged in confocal (red) and 3D tau-STED (green). Square indicates a single GATTA bead, shown in a larger view in confocal and STED channels on the right. Scale bars, 1 µm (left), 100 nm (inset, right). (B) Confocal and tau-STED line profiles through individual GATTA beads. Red line shows the mean (54 nm, n = 43 beads, gray lines) with Rayleigh criterion of 13%. (C) Average peak-to-peak distances calculated from line profiles of 50 ± 5 nm spaced GATTA beads imaged in 2D (0% Z-depletion, n = 29 beads) and 3D (20% Z-depletion, n = 43 beads, p = 0.0697, unpaired Student’s t test). (D, E) Representative confocal and 3D tau-STED images of dual-labeled GATTA bead nano-rulers and associated line profiles (n = 60 beads, gray lines). Beads labeled with Atto 647N are spaced at 140 ± 5 nm, while Atto 647N and Atto 594 beads are spaced at 70 ± 5 nm. Green and red lines represent means (Rayleigh criterion of 33%). Scale bar: 250 nm. (F) Representative image of 3D GATTA 90 ± 5 nm axially spaced Atto 647N-labeled nano ruler beads imaged in 3D tau-STED. The bottom image shows orthogonal xz views of beads # 1–4. Scale bar, 1 µm. (G) Line profiles of beads # 1–4 in the xz view (shown in F) demonstrating peak-to-peak separation of 90 to 160 nm. (H) Zoomed-in view of the bead # 4 and associated orthogonal (xy and xz) projections. Scale bar, 500 nm. (I, J) 3D Neurolucida 360 reconstruction of GATTA beads from F. Beads #3 and #4 are shown. Inset (J) shows zoomed-in view of bead # 4. (K) Average nearest-neighbor distances calculated from Neurolucida 360 reconstructions of 90 ± 5 nm axially spaced GATTA beads (d = 93.6 ± 2.9 nm, n = 133 beads). Mean (red line) with individual GATTA beads (dots) are shown. (L) Average volume of single GATTA beads calculated from 3D Neurolucida 360 reconstructions of 90 ± 5 nm axially spaced GATTA beads (v = 0.0012 ± 0.0001 µm3, n = 136 beads). Mean (red line) w [file pbio.3002649.s006.tif]

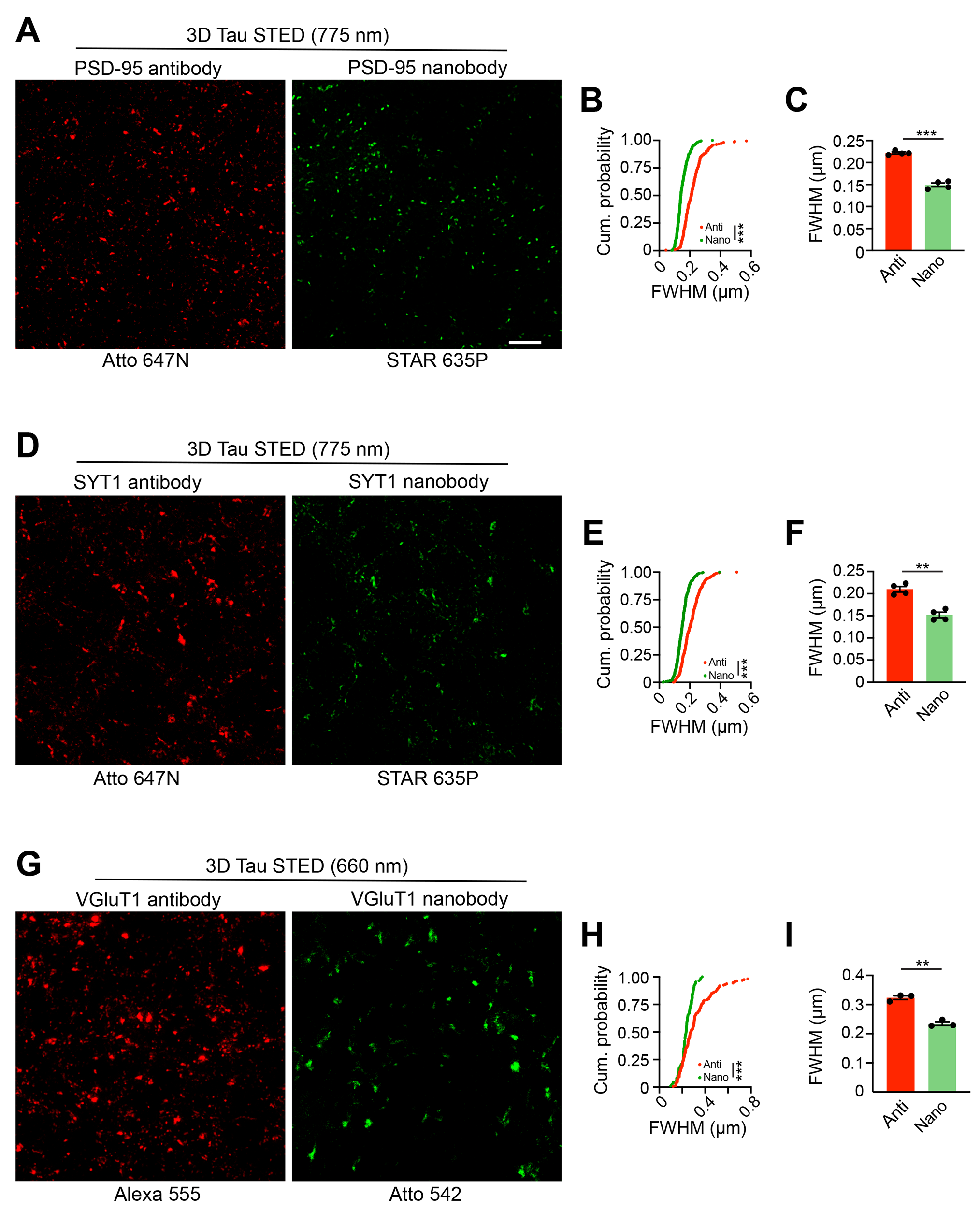

Supplement: S7 Fig — (A) 3D tau-STED images of PSD-95 clusters in DIV 21 cortical cultures stained with either a mouse primary and Atto 647N-conjugated secondary antibody (red) or a PSD-95-specific nanobody (Abberior STAR 635P, green). Images were acquired separately from sister cultures with the same settings. Scale bar for A, D, G: 3 µm. (B) Cumulative frequency distributions of FWHM for tau-STED-resolved PSD-95 nanoclusters labeled with antibody (red, n = 214) and nanobody (green, n = 275; ***p < 0.0001, Kolmogorov–Smirnov test). (C) Average FWHM per image for PSD-95 nanoclusters labeled with antibody and nanobody (n = 4 images, ***p < 0.0001, unpaired Student’s t test). (D) 3D tau-STED images of SYT1 clusters in DIV 21 cortical cultures stained with either a mouse primary and Atto 647N-conjugated secondary antibody (red) or a SYT1-specific nanobody (Abberior STAR 635P, green). Images were acquired separately from sister cultures using the same STED settings. (E) Cumulative frequency distributions of FWHM for tau-STED-resolved SYT1 nanoclusters labeled with antibody (red, n = 322) and nanobody (green, n = 333; ***p < 0.0001, Kolmogorov–Smirnov test). (F) Average FWHM per image for SYT1 nanoclusters labeled with antibody and nanobody (n = 4 images, **p = 0.0006, unpaired Student’s t test). (G) 3D tau-STED images of VGluT1 nanoclusters in DIV 21 cortical cultures labeled with either a mouse primary and Alexa 555-conjugated secondary antibody (red) or a VGluT1-specific nanobody (Atto 542, green). Images were acquired separately from sister cultures using the same STED settings. (H) Cumulative frequency distributions of FWHM for tau-STED-resolved VGluT1 nanoclusters labeled with antibody (red, n = 160) and nanobody (green, n = 86; ***p < 0.0001, Kolmogorov–Smirnov test). (I) Average FWHM per image for VGluT1 nanoclusters labeled with antibody and nanobody (n = 3 images, **p = 0.0006, unpaired Student’s t test). Bar graphs represent means ± SEM obtained from at least three different image [file pbio.3002649.s007.tif]

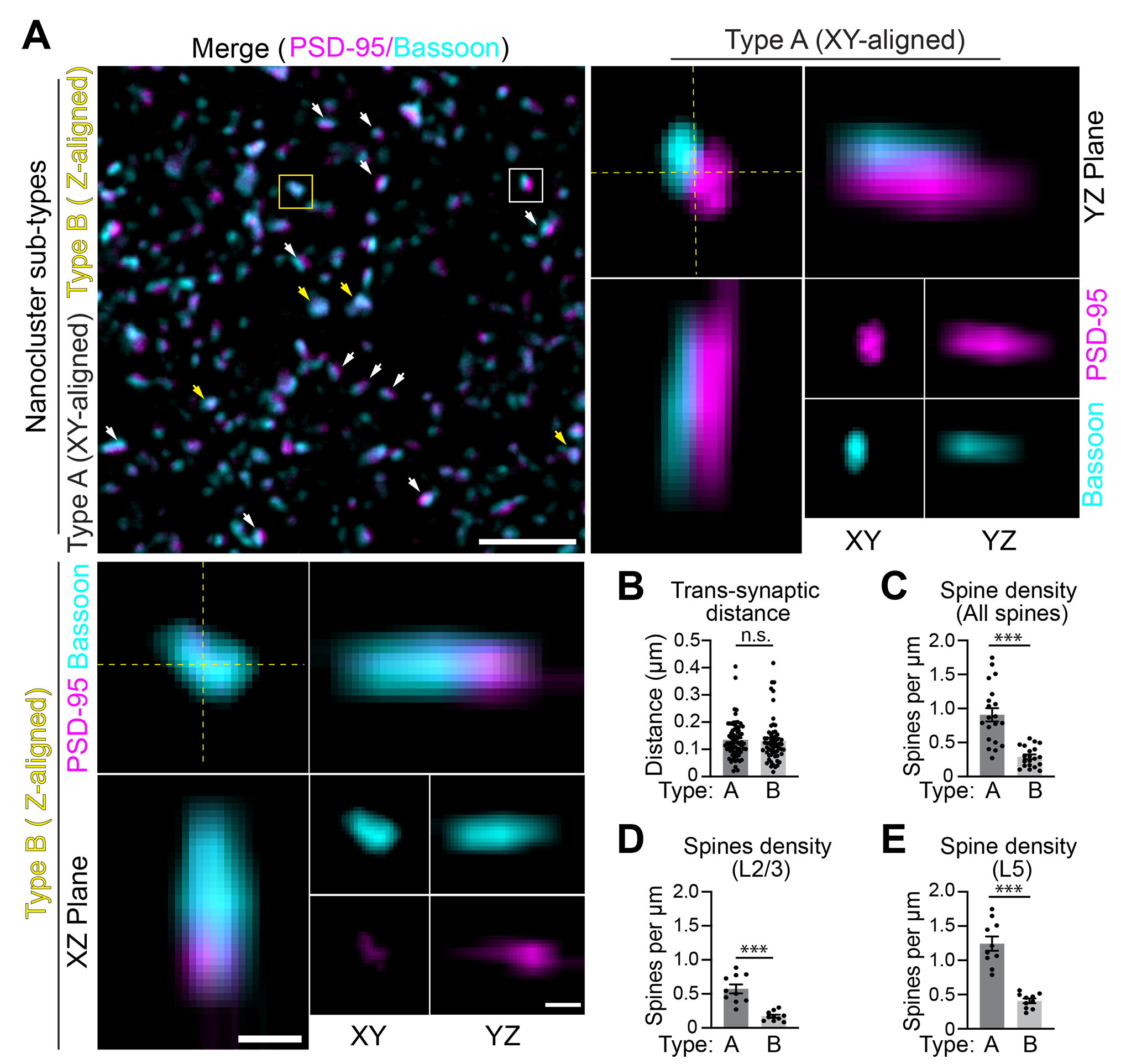

Supplement: S8 Fig — (A) A representative two-color STED image of PSD-95 (magenta) and Bassoon (cyan) nanoclusters in layer 5 (L5). White arrows indicate puncta aligned in the XY plane (Type A), while yellow arrows point to puncta aligned in the Z plane (Type B). Orthogonal views of Type A nanoclusters (white square) are shown on the right, and orthogonal views of Type B nanoclusters (yellow square) are displayed below. Scale bars: 2 µm, orthogonal views 200 nm. (B) Quantification of center-to-center distances between PSD-95 and Bassoon for Type A and Type B trans-synaptic nanoclusters (p = 0.8258, unpaired Student’s t test). Each dot represents an individual Type A (n = 72) or Type B (n = 59) trans-synaptic PSD-95/Bassoon nanocluster pair. (C) Densities of YFP-labeled spines on dendrites of L5 neurons, projecting in XY and Z orientations. Dots represent average spine densities for the indicated subtypes in apical (L2/3) and basal (L5) dendrites (n = 20 dendritic segments, ***p < 0.0001, unpaired Student’s t test). (D) Density of XY (Type A) and Z (Type B) projecting YFP-labeled spines along apical dendrites (Layers 2/3) from Thy-1-YFP-H mice. Dots represent average spine densities across 10 different dendritic segments (***p < 0.0001, unpaired Student’s t test). (E) Density of Type A and Type B projecting YFP-labeled spines along basal dendrites (Layer 5) from Thy-1-YFP-H mice. Dots represent average spine densities across 10 different dendritic segments (***p < 0.0001, unpaired Student’s t test). Bar graphs represent means ± SEM collected from two Thy-1-YFP-H mice. The spread of the data, indicated by dots on the bar graphs, is defined above. The source data for panels B-E can be found in S2 Table. (TIF) [file pbio.3002649.s008.tif]

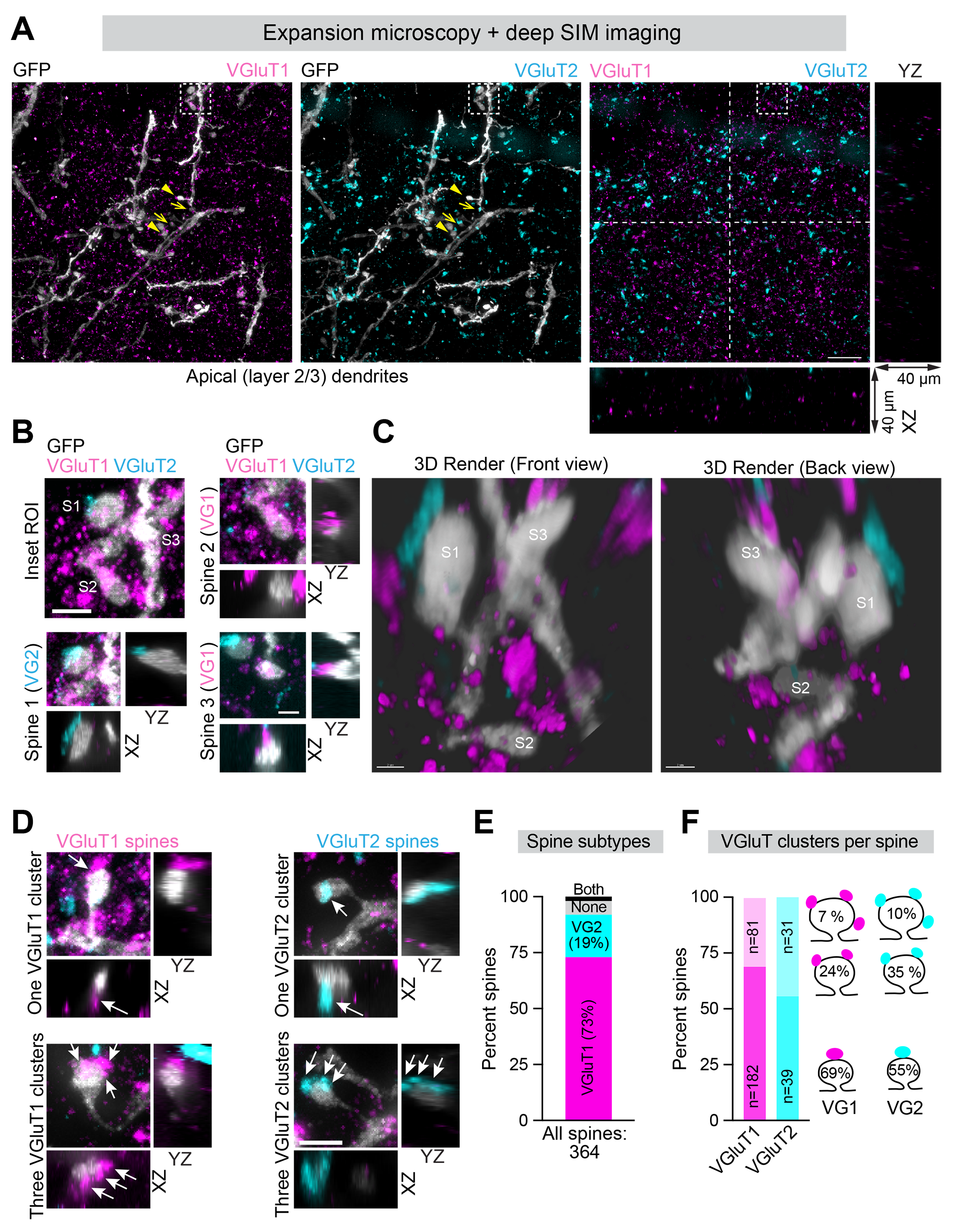

Supplement: S9 Fig — (A) A representative image of L5 pyramidal neuron apical dendrites in an expanded 5 µm cryosection from Thy-1-YFP H mouse S1 cortex imaged using deep SIM microscopy (Ex-SIM). Antibodies were used to recover and detect VGluT1 (magenta) and VGluT2 (cyan) after proteome expansion to assess their colocalization with dendritic spines (YFP, gray enhanced by GFP immunolabeling) in X, Y, and Z planes. Orthogonal views indicate the non-overlapping localization of VGluT1 and VGluT2 clusters in the Z plane. Scale bar (XY): 20 µm. (B) High-resolution images of three spines from the inset (square) in A. Expansion of samples in X, Y, and Z planes allowed unambiguous VGluT1 and VGluT2 assignment to individual dendritic spines. Scale bar: 5 µm (inset ROI), 2 µm (spines). (C) 3D rendering of spines in B confirms non-overlapping VGluT1 and VGluT2 localization to individual spines. Scale bars: 2 µm. (D) Discrete VGluT1 (yellow arrowheads in A) and VGluT2 (yellow arrows in A) clusters on individual spines using Ex-SIM. White arrows indicate discrete nanoclusters on YFP-labeled spines. Scale bar: 5 µm. (E) Fraction of spines receiving VGluT1 (n = 264 spines) or VGluT2 (n = 70 spines) input. Few spines (n = 7) had both VGluT1 and VGluT2 clusters and three spines did not colocalize with either VGluT1 or VGluT2. (F) Fraction of spines with one versus multiple (two and three or more) VGluT1 or VGluT2 clusters. The source data for panels E and F can be found in S2 Table. (TIF) [file pbio.3002649.s009.tif]

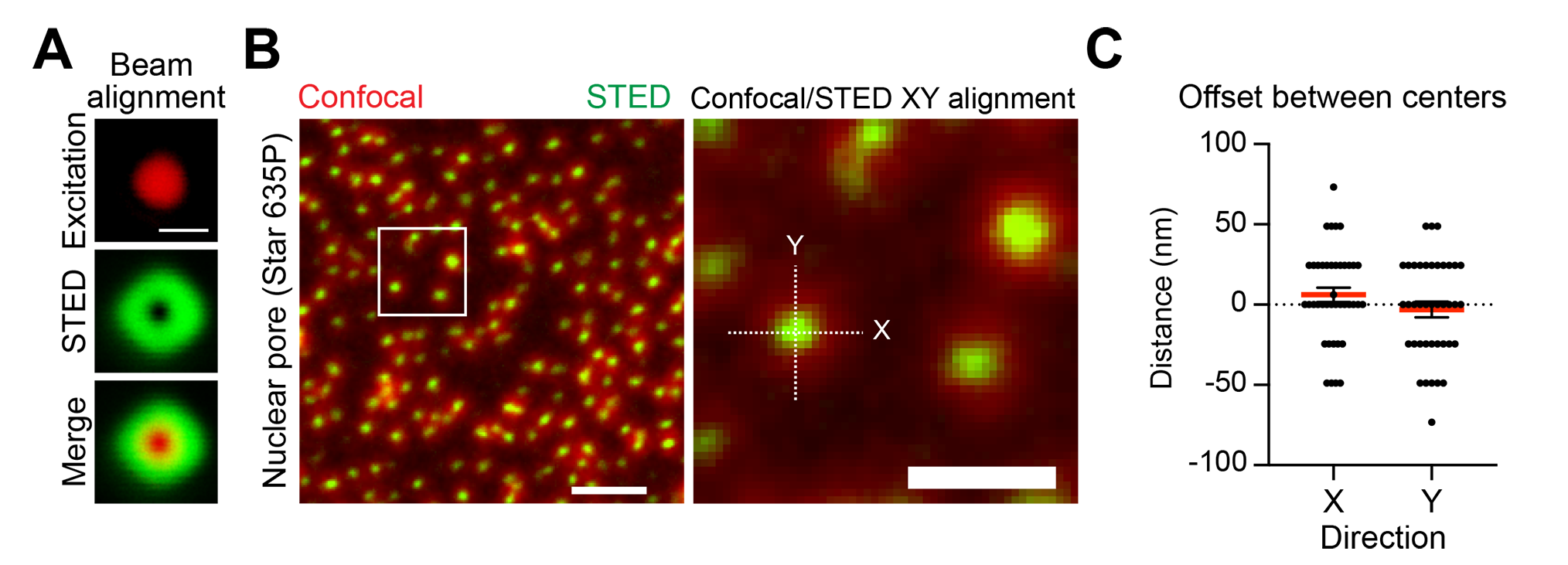

Supplement: S10 Fig — (A) Aligned confocal (red) and STED (green) beams visualized using light reflection from 80 nm gold particles. (B) Offset verification between confocal (red) and STED (green) signals using a standard Leica slide labeled with a nuclear pore antibody (Abberior STAR 635P) and imaged with a 100× 1.4 N/A STED objective. The square indicates a magnified view of the indicated clusters. The dotted lines show the direction of line profiles for offset measurement. Scale bars: 1 µm (left), 500 nm (right). (C) Average offsets between the centers of confocal and STED signals in the X (+6 nm, n = 40 clusters) and Y (−3 nm, n = 40 clusters) directions are below the STED resolution limit (~50 nm, see S6 Fig). The source data for the panel C can be found in S2 Table. (TIF) [file pbio.3002649.s010.tif]
