# Supplementary material for: Combining nanobody labeling with STED microscopy reveals input-specific and layer-specific organization of neocortical synapses
Source: PLoS Biol. 2025 Apr 4;23(4):e3002649. doi: 10.1371/journal.pbio.3002649 (PMC12002638; doi:10.1371/journal.pbio.3002649)
Supplement: S1 Table — (DOCX) [file pbio.3002649.s011.docx]

**Supplementary Table 1. Nanobodies and Antibodies**

| **Nanobodies and Primary Antibodies** | **Company** | **Reference #** | **RRID** | **Concentration** |
| --- | --- | --- | --- | --- |
| FluoTag-X2 anti-PSD-95 directly conjugated to Abberior STAR 635P | NanoTag Biotechnologies | # N3702 | 3076102 | Stock 2.5 µM, used at [1:300] |
| FluoTag-X2 anti-PSD95 directly conjugated to Abberior STAR 580 | NanoTag Biotechnologies | # N3702 | N/A | Stock 2.5 µM, used at [1:300] |
| FluoTag-X2 anti-VGluT1 directly conjugated to Atto 542 | NanoTag Biotechnologies | # N1602 | N/A | Stock 2.5 µM, used at [1:300] |
| FluoTag-X2 anti-Synaptotagmin 1 directly conjugated to AZDye 568 | NanoTag Biotechnologies | # N2302 | 3076136 | Stock 2.5 µM, used at [1:300] |
| Chicken anti-GFP | Abcam | # ab13970 | 300798 | Stock 10 mg/mL, used at [1:2000] |
| Mouse IgG1 anti-PSD-95 | Invitrogen | # MA1-046 | 2092361 | Stock 1 mg/mL, used at [1:500] |
| Mouse IgG2a anti-PSD-95 | NeuroMab | # 75-028 | 2292909 | Stock 1 mg/mL, used at [1:200] |
| Mouse IgG2b anti-Bassoon | NeuroMab | # 75-491 | 2716712 | Stock 1 mg/mL, used at [1:500] |
| Guinea pig anti-VGluT1 | Millipore | # AB5905 | 2301751 | Used at [1:4000] |
| Rabbit anti-VGluT2 | Synaptic Systems | # 135 408 | 2864778 | Stock 1 mg/mL, used at [1:1000] |
| Mouse IgG2a anti-Synaptotagmin 1 | Synaptic Systems | # 105 011 | 29274147 | Stock 1 mg/mL, used at [1:500] |
| **Secondary Antibodies** | **Company** | **Reference #** | **RRID** | **Concentration** |
| Goat anti-chicken (Alexa Fluor 488) | Jackson Immunoresearch | # 103-545-155 | 2337390 | Stock 1.5 mg/mL, used at [1:500] |
| Goat anti-guinea pig (Alexa Fluor 555) | Invitrogen | # A21435 | 2535856 | Stock 2 mg/mL, used at [1:500] |
| Goat anti-mouse IgG2a (Alexa Fluor 594) | Jackson Immunoresearch | # 115-585-206 | 2338886 | Stock 1.6 mg/mL, used at [1:500] |
| Goat anti-mouse IgG2b (Alexa Fluor 594) | Jackson Immunoresearch | # 115-585-207 | 2338887 | Stock 1.7 mg/mL, used at [1:500] |
| Goat anti-Guinea pig (Alexa Fluor 594) | Invitrogen | # A11076 | 2534120 | Stock 2 mg/mL, used at [1:500] |
| Goat anti-mouse IgG2a (ATTO 647N) | Rockland | # 610-156-041 | 2614871 | Stock 1 mg/mL, used at [1:500] |
| Goat anti-rabbit (ATTO 647N) | Rockland | # 611-156-122 | 10893043 | Stock 1 mg/mL, used at [1:500] |
| Goat anti rabbit (Alexa Fluor 790) | Jackson Immunoresearch | # 111-655-144 | 2338086 | Stock 1.5 mg/mL, used at [1:500] |
